#  Versatile routes for synthesis of diarylamines through acceptorless dehydrogenative aromatization catalysis over supported gold–palladium bimetallic nanoparticles†
†Electronic supplementary information (ESI) available. See DOI: 10.1039/c6sc04455g



**DOI:** 10.1039/c6sc04455g

**Published:** 2016-12-01

**Authors:** Kento Taniguchi, Xiongjie Jin, Kazuya Yamaguchi, Kyoko Nozaki, Noritaka Mizuno

**Affiliations:** a Department of Applied Chemistry , School of Engineering , The University of Tokyo , 7-3-1 Hongo, Bunkyo-ku , Tokyo 113-8656 , Japan . Email: tmizuno@mail.ecc.u-tokyo.ac.jp ; Email: kyama@appchem.t.u-tokyo.ac.jp ; Fax: +81-3-5841-7220; b Department of Chemistry and Biotechnology , School of Engineering , The University of Tokyo , 7-3-1 Hongo, Bunkyo-ku , Tokyo 113-8656 , Japan

## Abstract

Diarylamines are an important class of widely utilized chemicals, and development of diverse procedures for their synthesis is of great importance. Herein, we have successfully developed novel versatile catalytic procedures for the synthesis of diarylamines through acceptorless dehydrogenative aromatization. In the presence of a gold–palladium alloy nanoparticle catalyst (Au–Pd/TiO_2_), various symmetrically substituted diarylamines could be synthesized starting from cyclohexylamines. The observed catalysis of Au–Pd/TiO_2_ was heterogeneous in nature and Au–Pd/TiO_2_ could be reused several times without severe loss of catalytic performance. This transformation needs no oxidants and generates molecular hydrogen (three equivalents with respect to cyclohexylamines) and ammonia as the side products. These features highlight the environmentally benign nature of the present transformation. Furthermore, in the presence of Au–Pd/TiO_2_, various kinds of structurally diverse unsymmetrically substituted diarylamines could successfully be synthesized starting from various combinations of substrates such as (i) anilines and cyclohexanones, (ii) cyclohexylamines and cyclohexanones, and (iii) nitrobenzenes and cyclohexanols. The role of the catalyst and the reaction pathways were investigated in detail for the transformation of cyclohexylamines. The catalytic performance was strongly influenced by the nature of the catalyst. In the presence of a supported gold nanoparticle catalyst (Au/TiO_2_), the desired diarylamines were hardly produced. Although a supported palladium nanoparticle catalyst (Pd/TiO_2_) gave the desired diarylamines, the catalytic activity was inferior to that of Au–Pd/TiO_2_. Moreover, the activity of Au–Pd/TiO_2_ was superior to that of a physical mixture of Au/TiO_2_ and Pd/TiO_2_. The present Au–Pd/TiO_2_-catalyzed transformation of cyclohexylamines proceeds through complex pathways comprising amine dehydrogenation, imine disproportionation, and condensation reactions. The amine dehydrogenation and imine disproportionation reactions are effectively promoted by palladium (not by gold), and the intrinsic catalytic performance of palladium is significantly improved by alloying with gold. One possible explanation of the alloying effect is the formation of electron-poor palladium species that can effectively promote the β-H elimination step in the rate-limiting amine dehydrogenation.

## Abstract 2

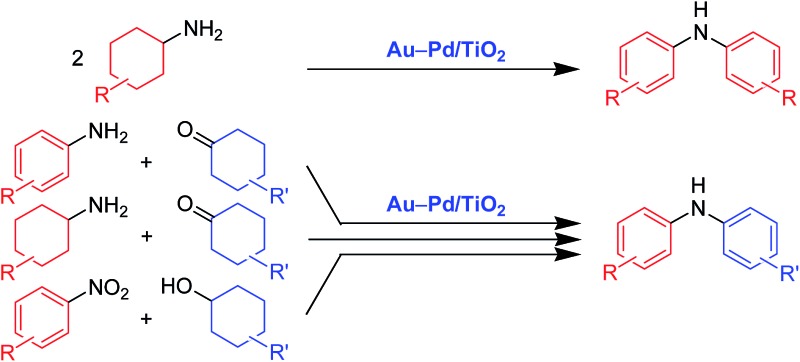
In the presence of Au–Pd/TiO_2_, various kinds of symmetrically and unsymmetrically substituted diarylamines could effectively be synthesized starting from various combinations of substrates.

## Introduction

**Fig. 1:**
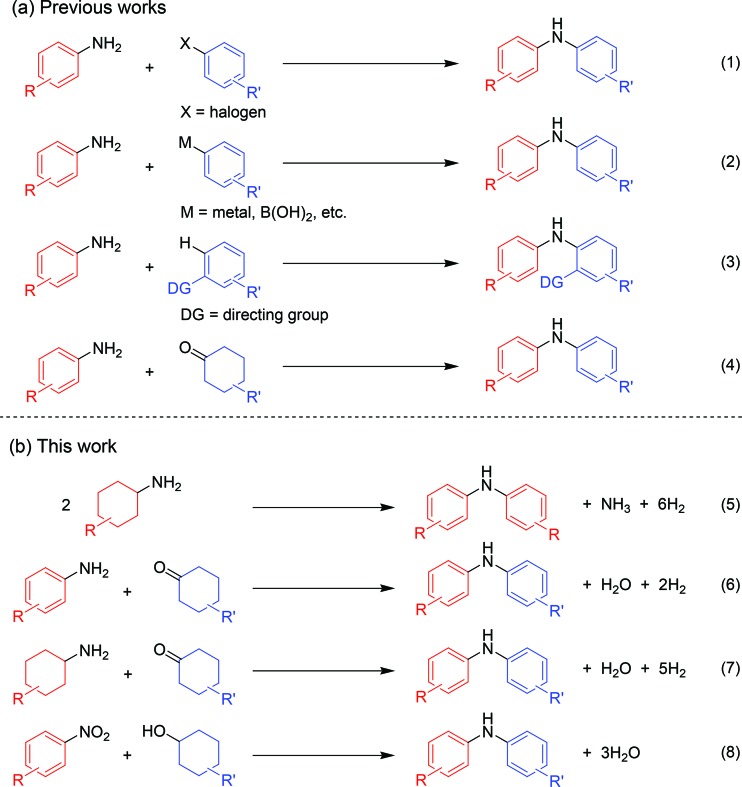
Synthetic procedures for diarylamines.

Diarylamines are very important compounds and core structural motifs frequently utilized for pharmaceuticals, agricultural chemicals, dyes, radical-trapping antioxidants, and electroluminescent materials.^1^ From the viewpoint of starting materials, the previously developed synthetic methods for diarylamines are roughly divided into four categories, namely (i) arylation of anilines using aryl halides (Fig. 1, eqn (1)),^2,3^ (ii) arylation of anilines using aryl metal or metalloid species (Fig. 1, eqn (2)),^4,5^ (iii) arylation of anilines involving directing-group assisted aromatic C–H bond activation (Fig. 1, eqn (3)),^6^ and (iv) dehydrogenative aromatization (Fig. 1, eqn (4)).^7^

The research groups of Buchwald and Hartwig have independently established the potent palladium-catalyzed cross-coupling reaction between anilines and aryl halides (Buchwald–Hartwig coupling, Fig. 1, eqn (1)).^2^ Arylation of ammonia using aryl halides is included in this category.^3^ As for arylation of anilines with aryl metal or metalloid species, the Chan–Lam coupling between anilines and arylboronic acids (or arylboronates) is undoubtedly one of the most powerful and versatile reactions,^4^ and the C–N cross-coupling reactions of anilines (or aniline surrogates) with aryl metal species, such as magnesium-, zinc-, nickel-, and gold-based reagents, have also been reported (Fig. 1, eqn (2)).^5^ These cross-coupling methodologies exhibit wide substrate scopes and are frequently utilized as reliable choices for diarylamine synthesis; however, they require pre-functionalized substrates, such as aryl halides and aryl metal species, which inevitably cause the concurrent formation of (super)stoichiometric amounts of waste. Although arylation of anilines with arenes through directing group-assisted aromatic C–H bond activation has recently been developed (Fig. 1, eqn (3)),^6^ most of the reported systems require (super)stoichiometric amounts of organic reagents or metal-based oxidants, and exhibit a limited scope of substrates.

Recently, dehydrogenative aromatization has emerged as an attractive strategy for synthesis of various aromatic compounds (Fig. 1, eqn (4)).^7,8^ For example, cyclohexanones and cyclohexanols are converted into the corresponding phenols,^8a–h^ and the reaction of cyclohexanones in the presence of amines gives the corresponding *N*-substituted anilines.^7^ Various kinds of oxidants (hydrogen acceptors), such as molecular oxygen, 1-octene, styrene, *tert*-butyl peroxybenzoate, 2,3-dichloro-5,6-dicyanobenzoquinone, and molecular iodine, have been utilized for dehydrogenative aromatization,^7,8^ and acceptorless dehydrogenative aromatization of cyclohexanones into phenols has also been reported.^8*f*^ The acceptorless dehydrogenation with a release of molecular hydrogen is more desirable reaction from the viewpoint of high atom economy, increasing environmental concerns, and hydrogen recycling.^9^ Meanwhile, we have recently reported gold–palladium bimetallic nanoparticles catalyze oxidative dehydrogenation reactions to afford *N*-substituted anilines^7*i*^ and phenols^8*g*^ using molecular oxygen as the sole oxidant.

**Fig. 2:**
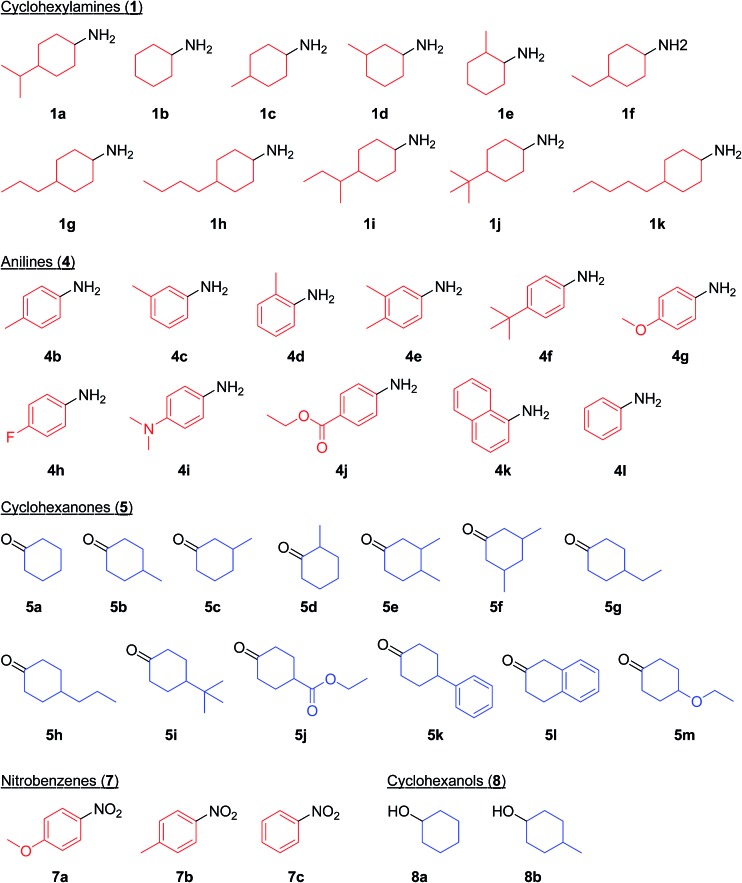
Substrates used in this study. **1a**, **1c–1k**, **5e**, **5f**, and **8b** are mixtures of *cis*–*trans* isomers.

As a continuation of our interest in the development of efficient catalytic dehydrogenation reactions, we report herein for the first time “acceptorless dehydrogenative aromatization” reactions to give symmetrically substituted diarylamines starting from cyclohexylamines (Fig. 1, eqn (5)), and unsymmetrically substituted diarylamines using various combinations of substrates such as anilines and cyclohexanones (Fig. 1, eqn (6)), cyclohexylamines and cyclohexanones (Fig. 1, eqn (7)), and nitrobenzenes and cyclohexanols (Fig. 1, eqn (8)). The substrates used in this study are summarized in Fig. 2. These reactions proceed without the use of oxidants under an Ar atmosphere and typically concurrently produce molecular hydrogen as a coproduct (no hydrogen evolution for eqn (8)). Additionally, catalysis using Au–Pd/TiO_2_ for the present dehydrogenative aromatization was truly heterogeneous and Au–Pd/TiO_2_ was easily recoverable and reusable without severe loss of catalytic performance.

## Results and discussion

### Effect of catalysts

**Fig. 3:**
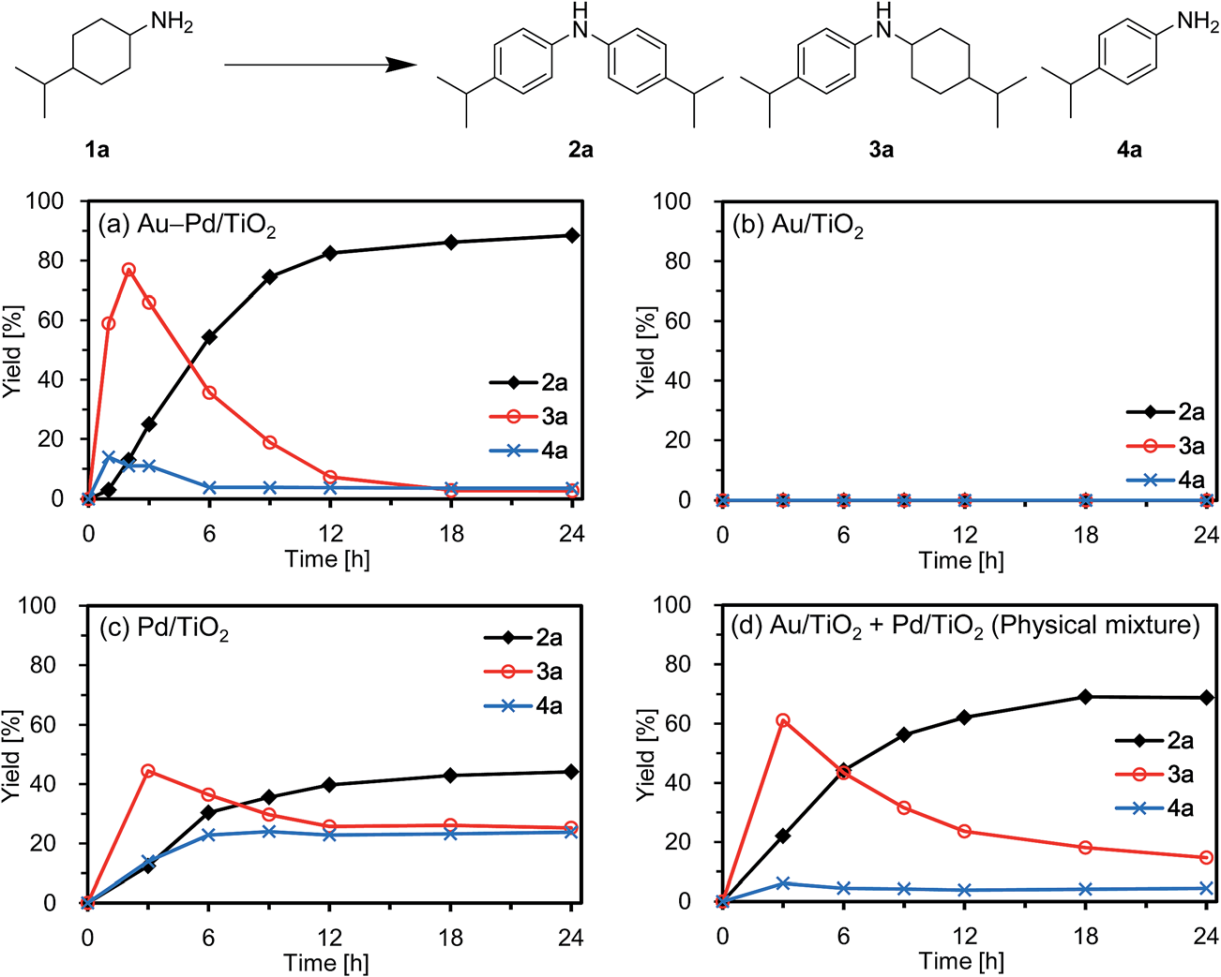
Reaction profiles for the acceptorless dehydrogenative aromatization of **1a** using various catalysts. Reaction conditions: catalyst (Au: 1.45 mol%, Pd: 1.05 mol%), **1a** (1.0 mmol), mesitylene (2 mL), 160 °C, Ar (1 atm). Yields were determined by GC analysis using *n*-hexadecane as an internal standard. (a) Au–Pd/TiO_2_. (b) Au/TiO_2_. (c) Pd/TiO_2_. (d) Au/TiO_2_ + Pd/TiO_2_ (physical mixture).

First, we carried out the dehydrogenative aromatization of 4-isopropylcyclohexylamine (**1a**) into 4-(1-methylethyl)-*N*-[4-(1-methylethyl)phenyl]benzenamine (**2a**) in the presence of various supported metal nanoparticles catalysts (gold, palladium, and gold–palladium on metal oxide supports; given in the format: metal/support, see in the Experimental section and ESI† for their preparation and characterization). The reactions were typically carried out at 160 °C in mesitylene under an Ar atmosphere (1 atm) without oxidants and additives. The reaction profiles are shown in Fig. 3. Gold–palladium bimetallic nanoparticles supported on TiO_2_ (Au–Pd/TiO_2_) showed excellent catalytic performance for acceptorless dehydrogenative aromatization. We prepared four kinds of Au–Pd/TiO_2_ catalysts with the different Au/Pd molar ratios (Au/Pd = 76/24, 58/42, 35/65, and 14/86) and carried out the reaction of **1a** using these four catalysts under the conditions described in Table S1.† Although the Au–Pd/TiO_2_ catalyst with the Au/Pd ratio of 58/42 (average particle size (*d*_av_): 3.3 nm, standard deviation (*σ*): 1.0 nm) gave the slightly better result than the others, the effect of the Au/Pd ratios was not so significant (Table S1, entries 1 and 9–11, ESI†). When using the best Au–Pd/TiO_2_ catalyst (Au/Pd = 58/42), the yield of **2a** reached 88% under the conditions described in Fig. 3 for 24 h (Fig. 3a, Table S1, entry 2, ESI†). In this reaction, we also confirmed the coproduction of *ca.* three equivalents (2.8 mmol) of hydrogen gas and *ca.* one-half equivalents (0.3 mmol) of ammonia gas with respect to **1a** (1.0 mmol). In contrast, the desired diarylamine **2a** was not produced at all in the presence of Au/TiO_2_ (*d*_av_: 1.9 nm, *σ*: 0.5 nm) (Fig. 3b, Table S1, entries 3 and 4, ESI†), thus showing that gold is completely inactive for the aromatization. When using Pd/TiO_2_ (*d*_av_: 2.0 nm, *σ*: 0.8 nm), **2a** was produced in 44% yield (Fig. 3c, Table S1, entry 6, ESI†), thus indicating that palladium is essential for the transformation and that Au–Pd/TiO_2_ has a superior catalytic activity relative to Pd/TiO_2_. A physical mixture of Au/TiO_2_ and Pd/TiO_2_ gave **2a** in 69% yield (Fig. 3d, Table S1, entry 8, ESI†), lower than that obtained with Au–Pd/TiO_2_.

High-angle annular dark-field scanning transmission electron microscopy (HAADF-STEM) and energy dispersive X-ray spectroscopy (EDS) analyses of Au–Pd/TiO_2_ revealed that random gold–palladium alloy nanoparticles were formed on TiO_2_ (Fig. S1, ESI†). The catalytic activity of the palladium species (based on the yield of **2a**) in Au–Pd/TiO_2_ (88% yield, Table S1, entry 2, ESI†) was at least twice that of Pd/TiO_2_ (44% yield, Table S1, entry 6, ESI†), clearly indicating that the intrinsic catalytic activity of palladium for the reaction was improved by alloying with gold. The enhancement in catalytic activity is likely due to an electronic ligand effect; such alloying effects are frequently observed for several oxidation reactions using gold–palladium alloy catalysts such as alcohol oxidation and hydrogen oxidation (H_2_O_2_ production).^10^ Also, in our previous study on Au–Pd/LDH-catalyzed (LDH = Mg–Al-layered double hydroxide) oxidative dehydrogenation of cyclohexanols and cyclohexanones to phenols using molecular oxygen as the oxidant, the improvement of palladium catalysis by alloying with gold was crucial.^8*g*^ The role of the metal species and the alloying effect will be discussed in additional detail below.

Next, the effect of supports was examined. Gold–palladium bimetallic nanoparticles, supported on TiO_2_, Al_2_O_3_, MgO, and CeO_2_, exhibited catalytic activities for the dehydrogenative aromatization of **1a**, and TiO_2_ was the best support among them (Table S1, entries 1 and 12–14, ESI†). When using SiO_2_ as the support, **2a** was hardly produced (Table S1, entry 15, ESI†), indicating that acidic supports are not suitable for the present transformation. Thus, we hereafter mainly utilize the most active Au–Pd/TiO_2_ catalyst (Au/Pd = 58/42) for further detailed investigations.

### Heterogeneous catalysis and catalyst reuse

**Fig. 4:**
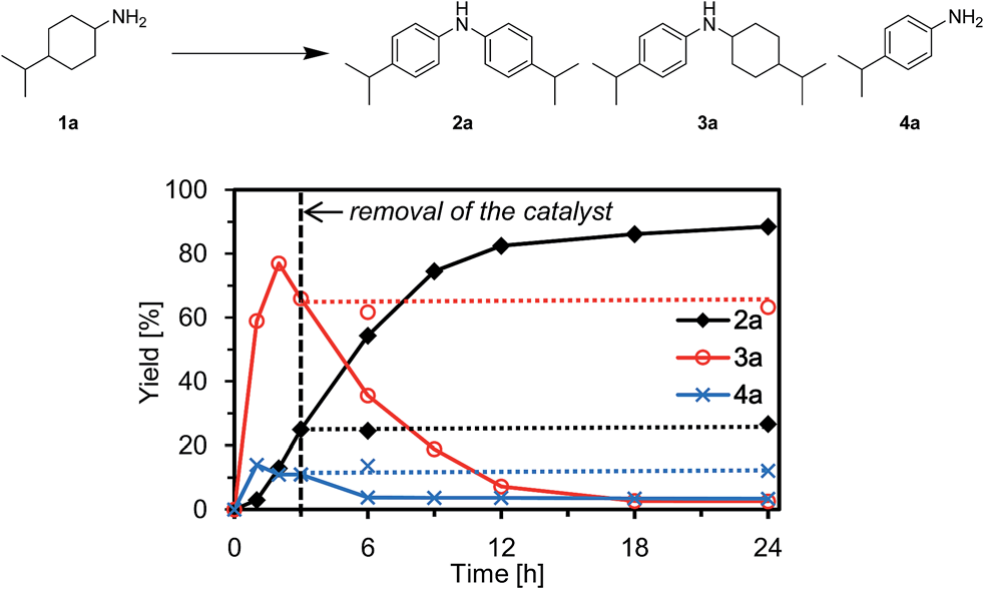
Effect of removal of Au–Pd/TiO_2_ (verification of heterogeneous catalysis). Reaction conditions: Au–Pd/TiO_2_ (Au: 1.45 mol%, Pd: 1.05 mol%), **1a** (1.0 mmol), mesitylene (2 mL), 160 °C, Ar (1 atm). Yields were determined by GC analysis using *n*-hexadecane as an internal standard. The arrow indicates the removal of Au–Pd/TiO_2_ by hot filtration.

To verify whether the observed catalysis with Au–Pd/TiO_2_ was truly heterogeneous, the following control experiments were carried out. The reaction of **1a** to produce the corresponding diarylamine **2a** was carried out under conditions described in Fig. 4, and Au–Pd/TiO_2_ was removed from the reaction mixture by hot filtration after 3 h, then the filtrate was heated at 160 °C. In this case, no further production of **2a** and no further consumption of 4-(1-methylethyl)-*N*-[4-(1-methylethyl)cyclohexyl]benzeneamine (**3a**) and 4-(1-methylethyl)benzenamine (**4a**) were observed, as shown in Fig. 4. In addition, it was confirmed by inductively coupled plasma atomic emission spectroscopy (ICP-AES) analysis that no gold and palladium species were detected in the filtrate.^11^ These results clearly indicate that the observed Au–Pd/TiO_2_-catalyzed dehydrogenative aromatization is truly heterogeneous in nature.^11^

**Fig. 5:**
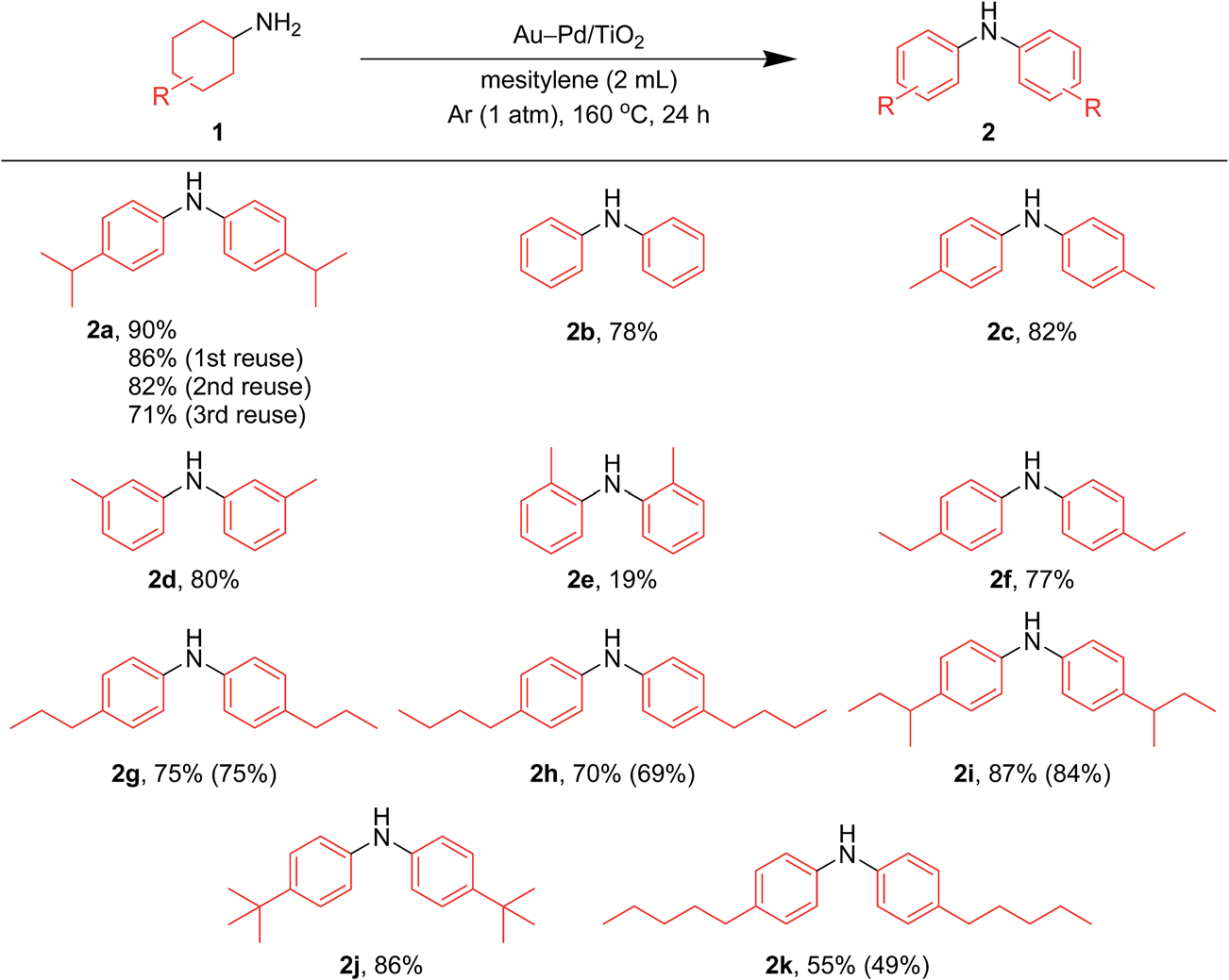
Scope of the Au–Pd/TiO_2_-catalyzed acceptorless dehydrogenative aromatization of cyclohexylamines. Reaction conditions: Au–Pd/TiO_2_ (Au: 1.45 mol%, Pd: 1.05 mol%), **1** (1.0 mmol), mesitylene (2 mL), 160 °C, Ar (1 atm), 24 h. Yields were determined by GC analysis using *n*-hexadecane as an internal standard. The values in the parentheses indicate the isolated yields.

After the reaction was complete, the Au–Pd/TiO_2_ could easily be retrieved from the reaction mixture by simple filtration with >90% recovery. We performed repeated reuse experiments for the transformation of **1a** under the standard reaction conditions using the retrieved catalyst. Although Au–Pd/TiO_2_ could be reused several times, the catalytic activity gradually decreased during the repeated reuse experiments; 90% yield of **2a** for the reaction of **1a** with the fresh catalyst, 86% yield for the first reuse, 82% yield for the second reuse, and 71% yield for the third reuse (Fig. 5). Transmission electron microscopy (TEM) analysis confirmed that the average particle size of gold–palladium alloy nanoparticles gradually increased during repeated reuse experiments; the average particle size in the fresh Au–Pd/TiO_2_ catalyst was 3.3 nm and increased to 4.4 nm after the third reuse experiment (Fig. S1, ESI†). HAADF-STEM and EDS analyses of Au–Pd/TiO_2_ used after the third reuse experiment revealed that random gold–palladium alloy nanoparticles were kept on TiO_2_ (Fig. S1, ESI†). Therefore, the gradual deactivation is likely caused by the increase in particle size.

### Substrate scope

**Fig. 6:**
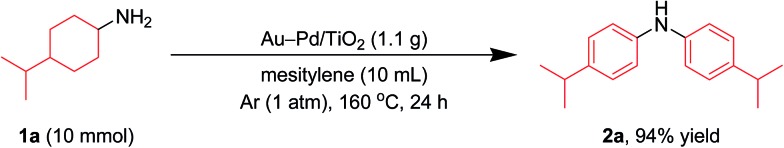
Larger scale acceptorless dehydrogenative aromatization of **1a**. Reaction conditions: Au–Pd/TiO_2_ (Au: 1.45 mol%, Pd: 1.05 mol%), **1a** (10.0 mmol), mesitylene (10 mL), 160 °C, Ar (1 atm), 24 h.

In this section, we examined the substrate scope for Au–Pd/TiO_2_-catalyzed dehydrogenative aromatization. The substrates used in this study are summarized in Fig. 2. As shown in Fig. 5, under optimized reaction conditions, various kinds of structurally diverse cyclohexylamines could be converted into the corresponding symmetrically substituted diarylamines in the presence of Au–Pd/TiO_2_. The reaction of cyclohexylamine and its derivatives with a methyl group at each position of the cyclohexane rings (**1a–1e**) efficiently proceeded to afford the corresponding diarylamines. Cyclohexylamines with other alkyl groups at their 4-position, such as ethyl (**1f**), *n*-propyl (**1g**), *n*-butyl (**1h**), *sec*-butyl (**1i**), *tert*-butyl (**1j**), and *n*-pentyl (**1k**) groups, could also be converted into the corresponding symmetrically substituted diarylamines. Notably, a larger scale reaction was also successful; even when a gram-scale (10 mmol-scale) acceptorless dehydrogenative aromatization of **1a** was performed, **2a** was obtained in 94% yield (Fig. 6).

**Fig. 7:**
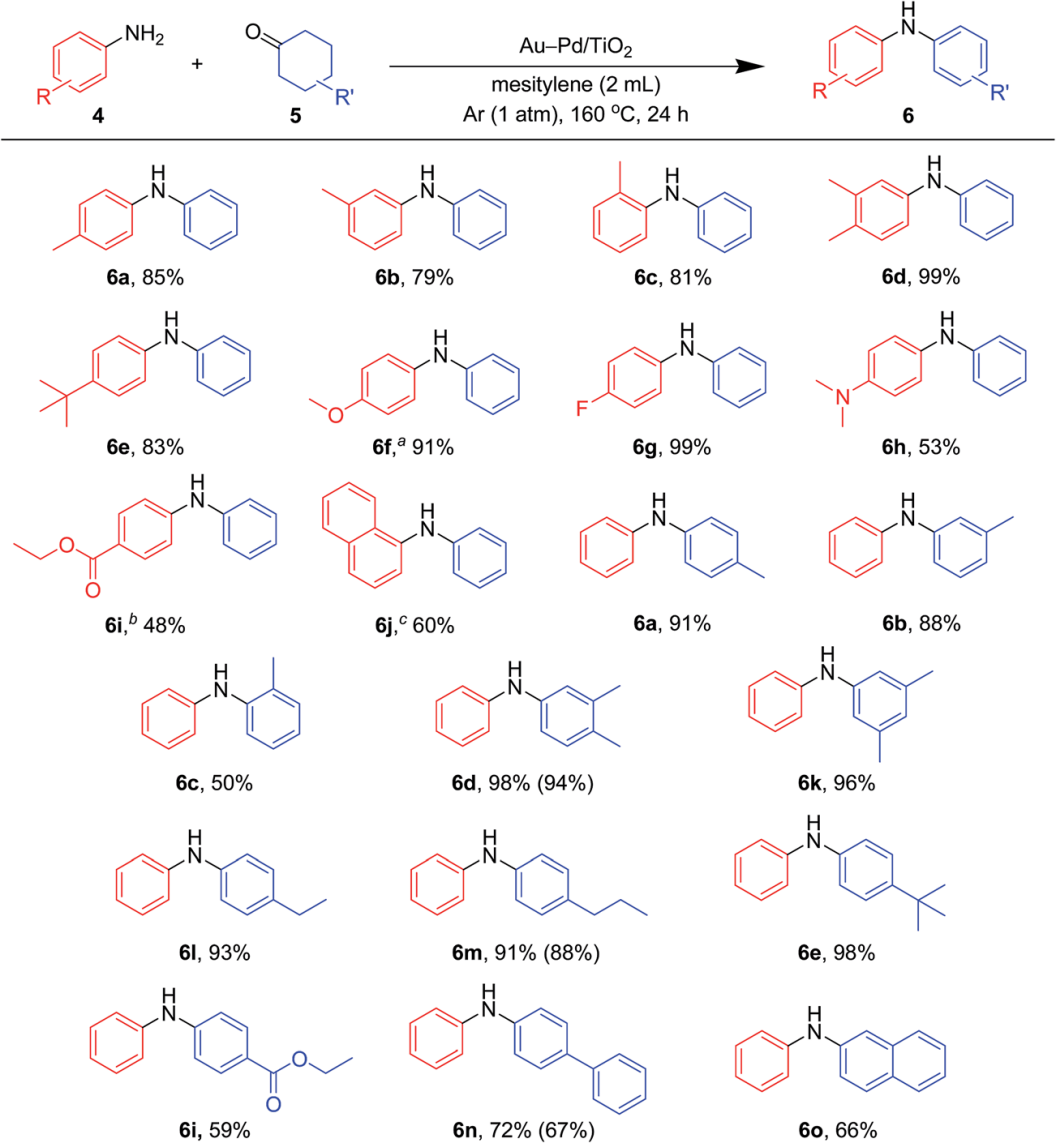
Scope of the Au–Pd/TiO_2_-catalyzed synthesis of diarylamines starting from cyclohexanones with anilines. Reaction conditions: Au–Pd/TiO_2_ (Au: 2.9 mol%, Pd: 2.1 mol%), **4** (0.5 mmol), **5** (0.5 mmol), mesitylene (2 mL), 160 °C, Ar (1 atm), 24 h. Yields were determined by GC analysis using *n*-hexadecane or biphenyl as an internal standard. The values in the parentheses indicate the isolated yields. ^*a*^Au–Pd/TiO_2_ (Au: 1.45 mol%, Pd: 1.05 mol%), 12 h. ^*b*^Au–Pd/TiO_2_ (Au: 0.73 mol%, Pd: 0.52 mol%). ^*c*^ **4k** (2.0 mmol), **5a** (0.5 mmol).

**Fig. 8:**
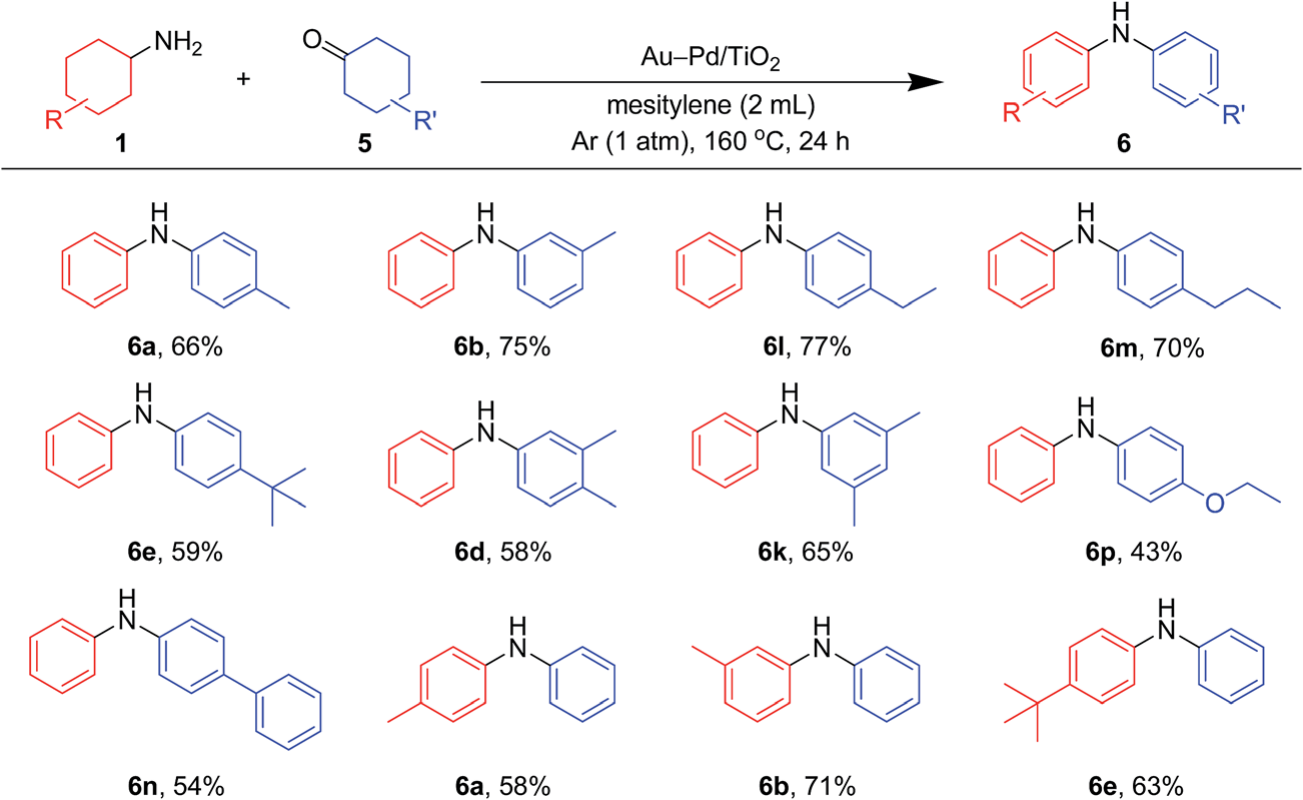
Scope of the Au–Pd/TiO_2_-catalyzed synthesis of diarylamines starting from cyclohexanones and cyclohexylamines. Reaction conditions: Au–Pd/TiO_2_ (Au: 2.9 mol%, Pd: 2.1 mol%), **1** (0.5 mmol), **5** (0.5 mmol), mesitylene (2 mL), 160 °C, Ar (1 atm), 24 h. Yields were determined by GC analysis using *n*-hexadecane as an internal standard.

**Fig. 9:**
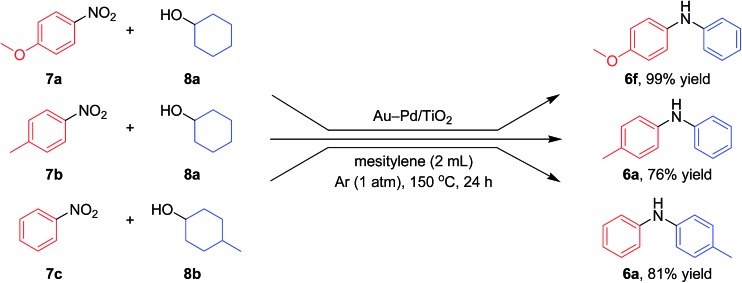
Scope of the Au–Pd/TiO_2_-catalyzed synthesis of diarylamines starting from tandem dehydrogenation aromatization of nitrobenzenes and cyclohexanols. Reaction conditions for the upper reaction: Au–Pd/TiO_2_ (Au: 5.8 mol%, Pd: 4.2 mol%), **7a** (0.25 mmol), **8a** (0.25 mmol), mesitylene (2 mL), 150 °C, Ar (1 atm), 24 h. Reaction conditions for the middle reaction: Au–Pd/TiO_2_ (Au: 2.9 mol%, Pd: 2.1 mol%), **7b** (0.5 mmol), **8a** (0.5 mmol), mesitylene (2 mL), 150 °C, Ar (1 atm). Reaction conditions for the bottom reaction: Au–Pd/TiO_2_ (Au: 2.9 mol%, Pd: 2.1 mol%), **7c** (0.5 mmol), **8b** (2.0 mmol), mesitylene (2 mL), 150 °C, Ar (1 atm). Yields were determined by GC analysis using *n*-hexadecane as an internal standard.

Various kinds of basic chemicals, such as cyclohexylamine, aniline, nitrobenzene, cyclohexanone, and cyclohexanol, are now industrially produced from benzene in large quantities.^12^ These basic chemicals and their derivatives are readily available, and thus the development of efficient catalytic transformations for production of profitable compounds directly utilizing them or their derivatives as starting materials is a very important subject. Therefore, to further explore the practical utility of the Au–Pd/TiO_2_-catalyzed acceptorless dehydrogenative aromatization system, we next attempted to synthesize unsymmetrically substituted diarylamines starting from various combinations of substrates such as (i) anilines and cyclohexanones (Fig. 7), (ii) cyclohexylamines and cyclohexanones (Fig. 8), and (iii) nitrobenzenes and cyclohexanols (Fig. 9).

As described in Fig. 7, various combinations of anilines and cyclohexanones (Fig. 2) successfully afforded the corresponding unsymmetrically substituted diarylamines in the presence of Au–Pd/TiO_2_. These reactions efficiently proceeded using equimolar mixtures of anilines and cyclohexanones. Anilines with alkyl groups (**4b–4f**) efficiently reacted with cyclohexanone (**5a**), giving the corresponding diarylamine derivatives. Anilines with various substituents, such as methoxy (**4g**), fluoro (**4h**), dimethylamino (**4i**), and ester (**4j**) groups, could be applied to the present system. 1-Naphthylamine (**4k**) could also be utilized in this reaction. Cyclohexanones possessing various substituents at each position, such as 4-methylcyclohexanone (**5b**), 3-methylcyclohexanone (**5c**), 2-methylcyclohexanone (**5d**), 3,4-dimethylcyclohexanone (**5e**), 3,5-dimethylcyclohexanone (**5f**), 4-ethylcyclohexanone (**5g**), 4-propylcyclohexanone (**5h**), 4-*tert*-butylcyclohexanone (**5i**), ethyl 4-oxocyclohexanonecarboxylate (**5j**), and 4-phenylcyclohexanone (**5k**), could successfully be utilized as coupling partners for aniline (**4l**) in the Au–Pd/TiO_2_-catalyzed system. The dehydrogenative aromatization of β-tetralone (**5l**) with **4l** afforded *N*-phenyl-β-naphthylamine (**6o**), which is one of the most important radical-trapping antioxidants.^13^

As shown in Fig. 8, unsymmetrically substituted diarylamines could also be synthesized starting from equimolar mixtures of cyclohexylamines and cyclohexanone (Fig. 2) through Au–Pd/TiO_2_-catalyzed acceptorless dehydrogenative aromatization. Through reactions with **1b**, cyclohexanones with various alkyl groups, such as 4-methyl (**5b**), 3-methyl (**5c**), 4-ethyl (**5g**), 4-propyl (**5h**), and 4-*tert*-butyl (**5i**), could be converted into the corresponding diarylamines. Disubstituted cyclohexanones, such as **5e** and **5f**, could also be used as substrates. The reaction of **5k** and 4-ethoxycyclohexanone (**5m**) with **1b** gave the corresponding diarylamines. Various cyclohexylamines, such as **1c**, **1d**, and **1j**, were also good coupling partners for **5a**, giving the corresponding diarylamine derivatives.

Notably, we could successfully synthesize unsymmetrically substituted diarylamines starting from nitrobenzenes (**7a–7c**) and cyclohexanols (**8a** and **8b**) in the presence of Au–Pd/TiO_2_ (Fig. 9). In this reaction, hydrogen formed in the alcohol dehydrogenation and aromatization steps is all effectively utilized for the selective six-electron reduction of nitrobenzenes. Therefore, the reaction efficiently proceeded using equimolar mixtures of nitrobenzenes and cyclohexanols. The reaction of nitrobenzenes and cyclohexanols to produce diarylamines has never been reported to date, to the best of our knowledge.

### Reaction pathways

**Fig. 10:**
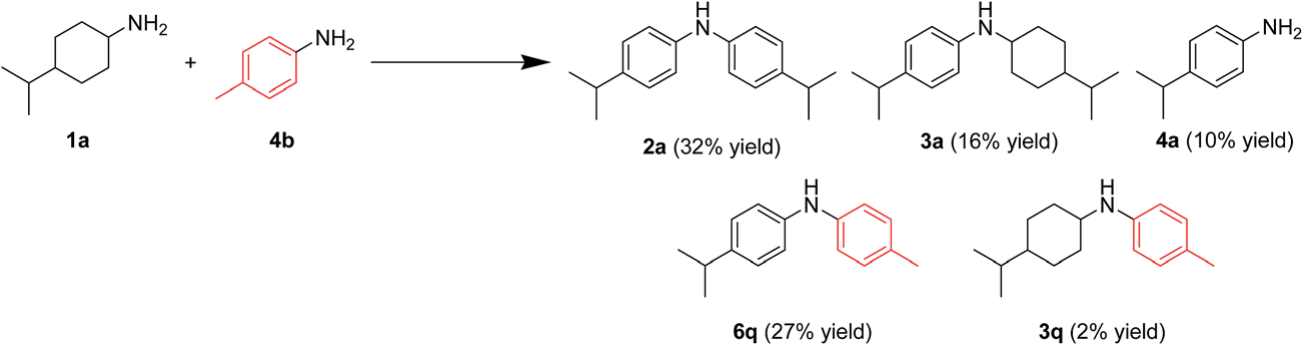
Acceptorless dehydrogenative aromatization of **1a** in the presence of **4b**. Reaction conditions: Au–Pd/TiO_2_ (Au: 1.45 mol%, Pd: 1.05 mol% to **1a**), **1a** (1.0 mmol), **4b** (1.0 mmol), mesitylene (2 mL), 160 °C, Ar (1 atm), 24 h. Yields were determined by GC analysis using *n*-hexadecane as an internal standard.

In this section, the reaction pathways for Au–Pd/TiO_2_-catalyzed transformation of **1a** to **2a** are discussed in detail. As shown in Fig. 3a, the reaction profile for the transformation of **1a** shows that **3a** and **4a** were initially produced followed by their conversion into the desired diarylamine **2a**. In a separate experiment, we confirmed that *N*-cyclohexylanilines were efficiently converted into the corresponding diarylamines under the same reaction conditions in the presence of Au–Pd/TiO_2_. Furthermore, when the Au–Pd/TiO_2_-catalyzed reaction of **1a** was carried out in the presence of *p*-toluidine (**4b**), significant amounts of amine–aniline cross-coupling products **6q** and **3q** were produced (Fig. 10). These results indicate that both *N*-cyclohexylanilines (**3**) and anilines (**4**) are possible intermediates for diarylamines.

**Fig. 11:**
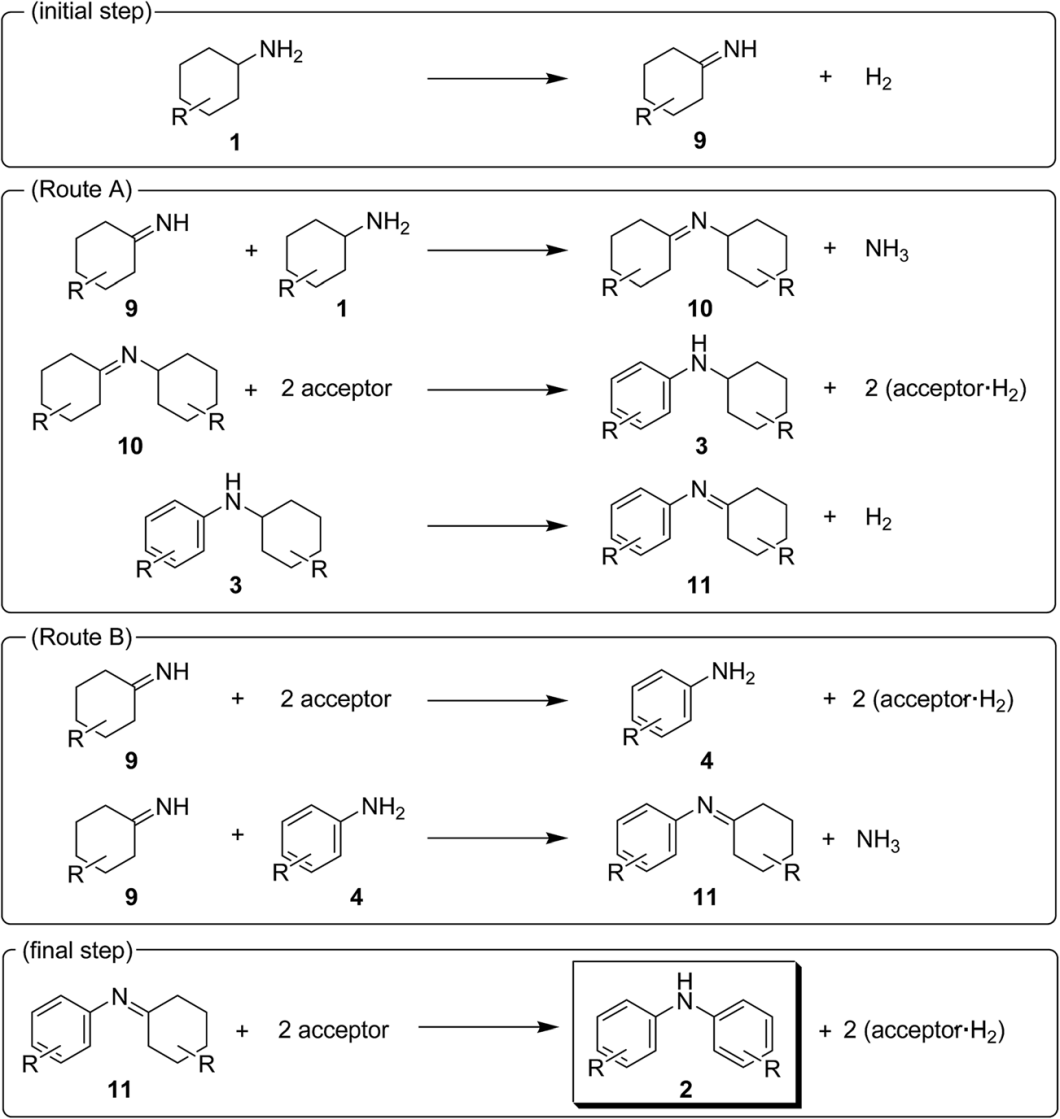
Proposed reaction pathways for the Au–Pd/TiO_2_-catalyzed acceptorless dehydrogenative aromatization of cyclohexylamines.

The present Au–Pd/TiO_2_-catalyzed dehydrogenative aromatization is likely initiated by the dehydrogenation of cyclohexylamine substrates (**1**) to cyclohexylimines (**9**). We hypothesize that the present diarylamine production is mainly made up of two routes (Fig. 11). In Route A, after the initial formation of **9**, the condensation of **9** and **1** gives the corresponding *N*-cyclohexylidenecyclohexanamines (**10**) followed by dehydrogenation to afford **3**. The dehydrogenation of **3** then takes place to give the corresponding *N*-phenylcyclohexylimines (**11**), followed by aromatization to afford the desired diarylamines **2**. In Route B, the further dehydrogenation of **9** produces **4**. After condensation of **9** and **4** aromatization occurs. The reaction profile in Fig. 3a shows that the concentration of **3a** was significantly higher than that of **4a** during the reaction. Therefore, we considered that Route A is the major pathway for the present diarylamine production. In addition, the dehydrogenation of **3** (*N*-arylated secondary amines) is much slower than that of **1** (primary amines), as can be seen from Fig. 3a.

**Fig. 12:**
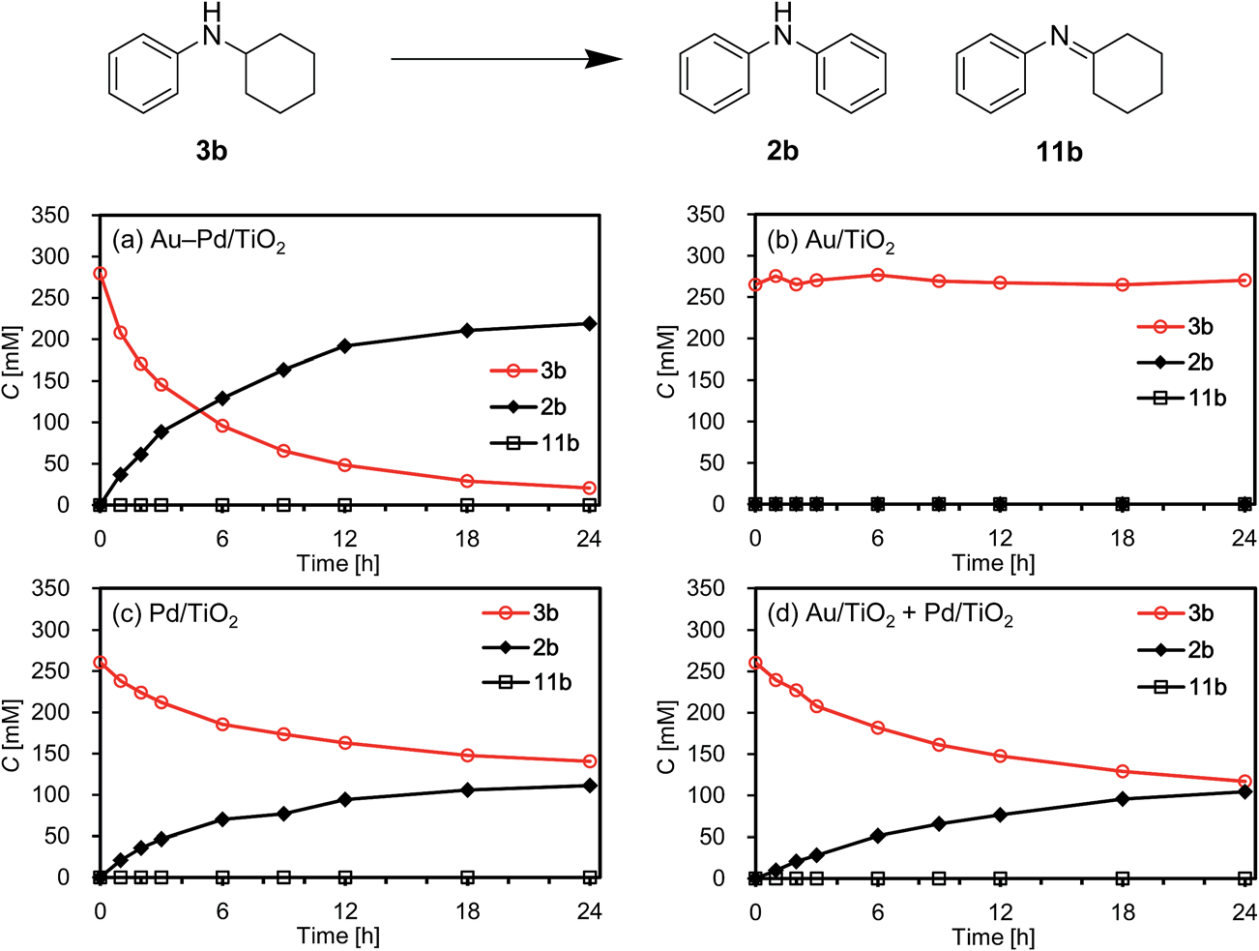
Reaction profiles for the acceptorless dehydrogenative aromatization of **3b**. Reaction conditions: catalyst (Au: 2.9 mol%, Pd: 2.1 mol%), **3b** (0.5 mmol), mesitylene (2 mL), 160 °C, Ar (1 atm). Yields were determined by GC analysis using *n*-hexadecane as an internal standard. (a) Au–Pd/TiO_2_. (b) Au/TiO_2_. (c) Pd/TiO_2_. (d) Au/TiO_2_ + Pd/TiO_2_ (physical mixture).

As above-mentioned, palladium was intrinsically effective for the transformation of cyclohexylamine **1a**, whereas gold was not effective (Fig. 3). In addition, the catalytic activity of palladium was significantly improved by alloying with gold (Fig. 3). In order to reveal the effect of the catalyst on the aromatization step in Route A in detail, we next carried out the reaction of *N*-cyclohexylaniline (**3b**) using Au–Pd/TiO_2_, Au/TiO_2_, Pd/TiO_2_, and a mixture of Au/TiO_2_ and Pd/TiO_2_. In the presence of Au–Pd/TiO_2_, the reaction efficiently proceeded to afford the corresponding diarylamine **2b** (Fig. 12a). When using Au/TiO_2_, almost no conversion of **3b** was observed (Fig. 12b), indicating gold is completely inactive for dehydrogenation of this type of amine. In the presence of Pd/TiO_2_, **2b** was produced in significant amounts (Fig. 12c), showing that palladium is essential for the transformation. The catalytic activity of a simple mixture of Au/TiO_2_ and Pd/TiO_2_ was almost the same as that of Pd/TiO_2_, and much lower than that of Au–Pd/TiO_2_ (Fig. 12d). Therefore, the effect of alloying palladium with gold is crucial for the transformation of **3**.

**Fig. 13:**
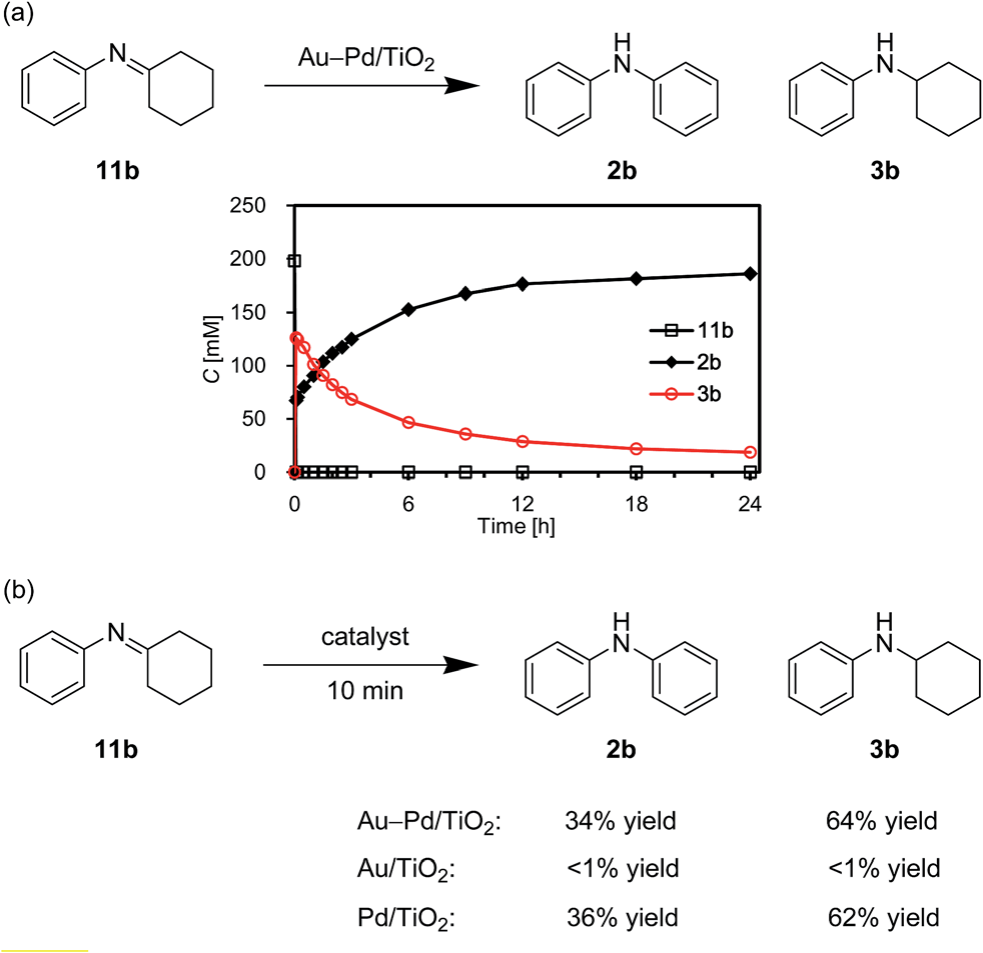
Acceptorless dehydrogenative aromatization of **11b** using various catalysts. (a) The reaction profile using Au–Pd/TiO_2_. (b) The reactions using various catalysts for 10 min. Reaction conditions: catalyst (Au: 2.9 mol%, Pd: 2.1 mol%), **11b** (0.4 mmol), mesitylene (2 mL), 160 °C, Ar (1 atm). Yields were determined by GC analysis using *n*-hexadecane as an internal standard.

We also performed the reaction of *N*-cyclohexylidenebenzenamine (**11b**). In the presence of Au–Pd/TiO_2_, **11b** was quantitatively converted into a *ca.* 1 : 2 mixture of **2b** and **3b** in the initial stage of the reaction (within 10 min) (Fig. 13a). After that, **11b** was hardly detected throughout the reaction (Fig. 13a). In addition, **11b** was not observed for the reaction starting from **3b** (Fig. 12a). These time courses clearly revealed that the desired diarylamine **2b** was produced through the disproportionation of **11b** without evolution of hydrogen gas and that the disproportionation was much faster than the dehydrogenation of **3b** to **11b**. Pd/TiO_2_ was also effective for the disproportionation, whereas Au/TiO_2_ was not (Fig. 13b), indicating that palladium is essential for the disproportionation.

On the basis of the above-mentioned pieces of experimental evidence, we concluded that Au–Pd/TiO_2_-catalyzed dehydrogenative aromatization proceeds through complex pathways composed of several amine dehydrogenation, imine disproportionation, and condensation reactions (Fig. 11). These amine dehydrogenation and imine disproportionation reactions are effectively promoted by palladium (not by gold), and the intrinsic catalytic performance of palladium is significantly improved by alloying with gold. The reaction is initiated by the acceptorless dehydrogenation of **1** to **9** with concomitant formation of hydrogen gas. In Route A (major route), the condensation of **9** and **1** takes place to give **10**. The disproportionation of **10** then takes place in which **10** itself, **9**, and **11** possibly act as the hydrogen acceptors, producing one molecule of **3** and two molecules of amines from three molecules of imines (one molecule of **10** and two molecules of **10**, **9** and/or **11**). The amines formed in the disproportionation step are again dehydrogenated to the corresponding imines by Au–Pd/TiO_2_. When **10** itself acts as the hydrogen acceptor for the disproportionation, dicyclohexylamine (**12**) is produced. We confirmed, in a separate experiment, that the desired diarylamine could efficiently be produced starting from **12** in the presence of Au–Pd/TiO_2_ (Fig. S2, ESI†). The dehydrogenation of **3** proceeds to give **11** followed by disproportionation to afford the desired diarylamines **2** with concomitant formation of amines, as with the reaction of **10**. In Route B (minor route), **9** is further dehydrogenated to **4**, possibly *via* disproportionation, which readily reacts with **9** to form **11**. Finally, the disproportionation of **11** affords the desired diarylamines **2**. Regardless of Route A or Route B, the reaction theoretically produces three equivalents of hydrogen gas and *ca*. one-half equivalents of ammonia gas with respect to **1**, which agrees well with the above-mentioned experimental result; the reaction of **1a** gave **2a** in 88% yield with concomitant formation of *ca.* three equivalents of hydrogen gas and *ca*. one-half equivalents of ammonia gas with respect to **1a** (Table S1, entry 2, ESI†).

In the reactions demonstrated in Fig. 7 and 8, the condensation of substrates (anilines + cyclohexanones or cyclohexylamines + cyclohexanones) initially takes place followed by the pathways indicated in Fig. 11. The reaction demonstrated in Fig. 9 is initiated by the dehydrogenation of cyclohexanols to cyclohexanones.^14^ Then, nitrobenzenes are reduced to the corresponding anilines by using the hydrogen formed (or transiently formed metal hydride species directly) in the initial alcohol dehydrogenation and dehydrogenative aromatization steps. The reaction then proceeds through the pathways shown in Fig. 11, giving the desired unsymmetrically substituted diarylamines **6** (Fig. S3, ESI†).

Quite recently, we reported an efficient aerobic oxidative dehydrogenation of cyclohexanols and cyclohexanones to phenols catalyzed by Au–Pd/LDH.^8*g*^ We revealed in the previous study that gold in Au–Pd/LDH plays an important assisting role on the β-H elimination from palladium–enolate species in the dehydrogenation of cyclohexanones to cyclohexenones by palladium, likely *via* an electronic ligand effect.^8*g*^ The greater electronegativity of gold compared to that of palladium has been reported to cause a net electron transfer from palladium to gold, thus resulting in the formation of more electron-poor palladium species.^10,15^ Consequently, the β-H elimination step using Au–Pd/LDH becomes more favorable in comparison to Pd/LDH.^8*g*^

We measured the XPS spectra of Au–Pd/TiO_2_ and Au/TiO_2_ around Au 4f components (Fig. S4, ESI†).^16^ The two peaks in each spectrum observed around 83 eV and 87 eV are attributed to Au 4f_7/2_ and 4f_5/2_, respectively. The Au 4f peaks of Au–Pd/TiO_2_ were observed at more negative binding energies in comparison with those of Au/TiO_2_. These significant negative shifts indicate the net electron-transfer from palladium to gold occurs to form electron-rich gold and electron-poor palladium species in Au–Pd/TiO_2_.^10,15^ As above-mentioned, the dehydrogenation of **3** is the rate-limiting reaction for the present Au–Pd/TiO_2_-catalyzed aromatization. The amine dehydrogenation possibly includes a β-H elimination step; it has been reported that the dehydrogenation of amines on the surface of palladium-based nanoparticles proceeds through the formation of palladium–amide species followed by β-H elimination.^17^ At this stage, our one possible explanation of the alloying effect is the formation of electron-poor palladium species by alloying with gold that can effectively promote the β-H elimination step in the rate-limiting amine dehydrogenation.

## Conclusion

We have successfully developed novel widely applicable synthetic procedures for formation of diarylamines. In the presence of a supported gold–palladium alloy nanoparticle catalyst (Au–Pd/TiO_2_), various kinds of structurally diverse symmetrically and unsymmetrically substituted diarylamines could be synthesized through acceptorless dehydrogenative aromatization starting from various substrate combinations. The observed catalysis was truly heterogeneous, and the Au–Pd/TiO_2_ catalyst could be reused. Owing to the practical and environmentally benign nature, we hope that the catalytic transformations developed in this study will find wide applications for the synthesis of diarylamine derivatives and their related compounds.

## Experimental section

### Instruments and reagents

GC analyses were performed on Shimadzu GC-2014 equipped with a flame ionization detector (FID) using a capillary column (InertCap5) for liquid phase analysis and Shimadzu GC-8A equipped with a thermal conductivity detector (TCD) using a glass tube column (5 Å molecular sieve) for gas phase analysis. GC-MS spectra were recorded on Shimadzu GCMSQP2010 equipped with a capillary column (InertCap5) at an ionization voltage of 70 eV. Liquid-state ^1^H and ^13^C NMR spectra were recorded on JEOL JNM-ECA 500 at 500 and 125 MHz, respectively, with TMS as an internal standard (*δ* = 0 ppm). ICP-AES analyses were performed on Shimadzu ICPS-8100. TEM measurements were performed on JEOL JEM-2010HC. HAADF-STEM and EDS images were obtained using a JEOL JEM-ARM 200F operating at 200 kV. TEM and STEM samples were prepared by placing a drop of the suspension on carbon-coated Cu grids and then dried in air. XPS analyses were performed using a JEOL JPS-9000 under Mg Kα radiation (*hν* = 1253.6 eV, 8 kV, 10 mA). The peak positions were calibrated with respect to the Ti 2p_3/2_ peak (459.4 eV).^18^ TiO_2_ (BET surface area: 316 m^2^ g^–1^, cat. no. ST-01, Ishihara Sangyo Kaisya), SiO_2_ (BET surface area: 274 m^2^ g^–1^, cat. no. CARiACT Q-10 (75–150 µm), Fuji Silysia Chemical Ltd.), Al_2_O_3_ (BET surface area: 160 m^2^ g^–1^, cat. no. KHS-24, Sumitomo Chemical), CeO_2_ (BET surface area: 111 m^2^ g^–1^, cat. no. 544841-25G, Aldrich), and MgO (BET surface area: 36 m^2^ g^–1^, cat. no. NO-0012-HP, Ionic Liquids Technologies), were commercially available. Solvents and substrates were obtained from Kanto Chemical, TCI, Wako, or Aldrich (reagent grade), and purified prior to use, if necessary.^19^

### Preparation of catalysts

Au–Pd/TiO_2_ (Au/Pd = 58/42) was prepared as follows. First, an aqueous solution of HAuCl_4_·4H_2_O (5.0 mM), PdCl_2_ (3.3 mM), and KCl (two equivalents with respect to PdCl_2_, 6.7 mM) containing TiO_2_ (2.0 g) was vigorously stirred at room temperature for 15 min. Then, the pH of the solution was adjusted to 10.0 using an aqueous solution of NaOH (1.0 M, *ca.* 1.5 mL). The resulting slurry was then further stirred for 24 h at room temperature giving 2.0 g of the hydroxide precursor. By the reduction of the hydroxide precursor using hydrogen (1 atm) at 150 °C for 30 min, the supported gold–palladium bimetallic nanoparticle catalyst Au–Pd/TiO_2_ was obtained. The contents of gold and palladium in Au–Pd/TiO_2_ were 0.134 mmol g^–1^ and 0.095 mmol g^–1^, respectively (determined by ICP-AES), and the average particle size (*d*_av_) was 3.3 nm (standard deviation (*σ*): 1.0 nm). Au–Pd/TiO_2_ (Au/Pd = 76/24, Au: 0.150 mmol g^–1^, Pd: 0.046 mmol g^–1^), Au–Pd/TiO_2_ (Au/Pd = 35/65, Au: 0.078 mmol g^–1^, Pd: 0.144 mmol g^–1^), Au–Pd/TiO_2_ (Au/Pd = 14/86, Au: 0.031 mmol g^–1^, Pd: 0.188 mmol g^–1^), and other supported catalysts were prepared essentially by the same manner as that for Au–Pd/TiO_2_ (Au/Pd = 58/42) (for the preparation of Au–Pd/MgO, an aqueous solution of NaOH was not used); Au/TiO_2_ (Au: 0.149 mmol g^–1^, *d*_av_: 1.9 nm, *σ*: 0.5 nm), Pd/TiO_2_ (Pd: 0.250 mmol g^–1^, *d*_av_: 2.0 nm, *σ*: 0.8 nm), Au–Pd/SiO_2_ (Au: 0.026 mmol g^–1^, Pd: 0.048 mmol g^–1^, *d*_av_: 6.4 nm, *σ*: 7.7 nm), Au–Pd/Al_2_O_3_ (Au: 0.111 mmol g^–1^, Pd: 0.090 mmol g^–1^, *d*_av_: 3.7 nm, *σ*: 1.1 nm), Au–Pd/CeO_2_ (Au: 0.128 mmol g^–1^, Pd: 0.086 mmol g^–1^, *d*_av_: 3.2 nm, *σ*: 1.1 nm), and Au–Pd/MgO (Au: 0.074 mmol g^–1^, Pd: 0.062 mmol g^–1^, *d*_av_: 5.1 nm, *σ*: 1.9 nm).

### Catalytic reactions

Catalytic reactions were typically carried out according to the following procedure. Into a Schlenk flask reactor (volume: *ca.* 20 mL) were successively placed Au–Pd/TiO_2_ (Au: 1.45 mol%, Pd: 1.05 mol% with respect to the cyclohexylamine), cyclohexylamine (**1**, 1.0 mmol), *n*-hexadecane (0.1 mmol, internal standard), mesitylene (2 mL), and a Teflon-coated magnetic stir bar, and then the mixture was stirred at 160 °C under Ar (1 atm). Substrate conversions and product yields were determined by GC analysis using *n*-hexadecane as an internal standard. As for isolation of the products, the internal standard was not used. After the reaction, the catalyst was removed by simple filtration (>90% catalyst recovery) and the filtrate was concentrated by evaporation of the mesitylene solvent. The crude product was subjected to column chromatography on silica gel (typically using chloroform/hexane as an eluent), giving the pure diarylamine product. The products were confirmed by comparison of their GC retention times, GC-MS spectra, and/or NMR (^1^H and ^13^C) spectra with those of the authentic data. The detection of hydrogen and ammonia in the gas-phase was carried out with GC (with a TCD detector) and GC-MS analyses. The quantification of hydrogen gas formation was performed by measurement of the evolved gas volume after ammonia trapping using aqueous HCl, and the quantification of ammonia formation was performed by titration of the aqueous HCl solution using aqueous NaOH. As for the reuse experiment, Au–Pd/TiO_2_ was retrieved by filtration and washed with a mixed solvent of methanol and dichloromethane. The retrieved Au–Pd/TiO_2_ was activated at 150 °C in 1 atm of hydrogen prior to being used for the reuse experiment.

## References

[cit1] (a) Lawrence S. A., Amines: Synthesis Properties and Applications, Cambridge University Press, Cambridge, 2004.

[cit2] Paul F., Patt J., Hartwig J. F. (1994). J. Am. Chem. Soc..

[cit3] Shen Q., Hartwig J. F. (2006). J. Am. Chem. Soc..

[cit4] Chan D. M. T., Manaco K. L., Wang R. P., Winters M. P. (1998). Tetrahedron Lett..

[cit5] Cloutier J.-P., Vabre B., Moungang-Soume B., Zargarian D. (2015). Organometallics.

[cit6] Kim H., Shin K., Chang S. (2014). J. Am. Chem. Soc..

[cit7] Girard S. A., Hu X., Knauber T., Zhou F., Simon M.-O., Deng G.-J., Li C.-J. (2012). Org. Lett..

[cit8] Izawa Y., Pun D., Stahl S. S. (2011). Science.

[cit9] Dobereiner G. E., Crabtree R. H. (2010). Chem. Rev..

[cit10] Nishimura S., Yakita Y., Katayama M., Higashimine K., Ebitani K. (2013). Catal. Sci. Technol..

[cit11] Sheldon R. A., Wallau M., Arends I. W. C. E., Schuchardt U. (1998). Acc. Chem. Res..

[cit12] (a) Weissermel K. and Arpe H.-J., Industrial Organic Chemistry, Wiley-VCH, Weinheim, 4th edn, 2003.

[cit13] Brownlie I. T., Ingold K. U. (1966). Can. J. Chem..

[cit14] It has been reported that Au–Pd alloy nanoparticle catalysts are effective for alcohol dehydrogenation.10 In addition, we have reported that Au–Pd alloy nanoparticle catalysts showed high performance for the dehydrogenation of cyclohexanols to cyclohexanones.8*g*

[cit15] Edwards J. K., Freakley S. J., Carley A. F., Kiely C. J., Hutchings G. J. (2014). Acc. Chem. Res..

[cit16] We also attempted to confirm the peak shifts of Pd 3d components in Au–Pd/TiO_2_. However, it was very difficult to confirm that because the peak due to Pd 3d_5/2_ was overlapped with that of Au 4d_5/2_ and the intensity of the peak attributed to Pd 3d_3/2_ was very low

[cit17] Furukawa S., Suga A., Komatsu T. (2014). Chem. Commun..

[cit18] (b) Chastain J., Handbook of X-ray Photoelectron Spectroscopy, Perkin-Elmer Corporation, Physical Elecronics Division, 1992.

[cit19] Armarego W. L. F. and Chai C. L. L., Purification of Laboratory Chemicals, Butterworth-Heinemann, Oxford, 5th edn, 2003.

