# Supplementary material for: Versatile routes for synthesis of diarylamines through acceptorless dehydrogenative aromatization catalysis over supported gold–palladium bimetallic nanoparticles
Source: Chem Sci. 2016 Dec 1;8(3):2131–42. doi: 10.1039/c6sc04455g (PMC5407272; doi:10.1039/c6sc04455g)
Supplement: Supplementary file 1 [file SC-008-C6SC04455G-s001.pdf]

Electronic Supplementary Material (ESI) for Chemical Science.
This journal is © The Royal Society of Chemistry 2016

*Electronic Supplementary Information (ESI) for*

# Versatile routes for synthesis of diarylamines through acceptorless dehydrogenative aromatization catalysis over supported gold–palladium bimetallic nanoparticles

Kento Taniguchi,[a] Xiongjie Jin,[a] Kazuya Yamaguchi,*[a] Kyoko Nozaki[b] and Noritaka Mizuno*[a]

[a] *Department of Applied Chemistry, School of Engineering, The University of Tokyo, 7-3-1 Hongo, Bunkyo-ku, Tokyo 113-8656, Japan. E-mail: tmizuno@mail.ecc.u-tokyo.ac.jp; kyama@appchem.t.u-tokyo.ac.jp; Fax: +81-3-5841-7220*

[b] *Department of Chemistry and Biotechnology, School of Engineering, The University of Tokyo, 7-3-1 Hongo, Bunkyo-ku, Tokyo 113-8656, Japan.*

Compound data of symmetrically substituted diarylamines (**2**)

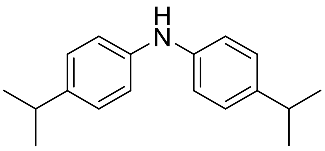

**2a (CAS registry number: 63451-41-2)**

**4-(1-Methylethyl)-*N*-[4-(1-methylethyl)phenyl]benzenamine (2a**; synthesized from **1a).** MS (70 eV, EI): *m/z* (%): 253 (50) [$M^+$], 254 (11), 239 (21), 238 (100), 222 (10), 112 (8).

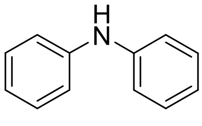

**2b (CAS registry number: 122-39-4)**

***N*-Phenylbenzenamine (2b**; synthesized from **1b).** MS (70 eV, EI): *m/z* (%): 169 (100) [$M^+$], 170 (14), 168 (54), 167 (28), 166 (5), 141 (5), 84 (12), 77 (8), 66 (6), 65 (6), 51 (9).

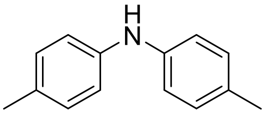

**2c (CAS registry number: 620-93-9)**

**4-Methyl-*N*-(4-methylphenyl)benzenamine (2c**; synthesized from **1c).** MS (70 eV, EI): *m/z* (%): 197 (100) [$M^+$], 198 (17), 196 (43), 182 (5), 181 (9), 180 (10), 97 (7), 91 (13), 77 (5).

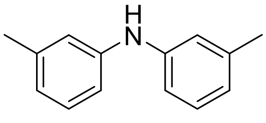

**2d (CAS registry number: 626-13-1)**

**3-Methyl-*N*-(3-methylphenyl)benzenamine (2d**; synthesized from **1d).** MS (70 eV, EI): *m/z* (%): 197 (100) [$M^+$], 198 (15), 196 (16), 182 (9), 181 (19), 180 (9), 167 (9), 97 (7), 91 (7), 79 (5), 77 (7), 65 (7).

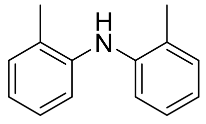

**2e (CAS registry number: 617-00-5)**

**2-Methyl-*N*-(2-methylphenyl)benzenamine (2e**; synthesized from **1e).** MS (70 eV, EI): *m/z* (%): 197 (100) [$M^+$], 198 (15), 196 (13), 182 (25), 181 (11), 180 (21), 167 (11), 104 (20), 106 (15), 97 (6), 93 (7), 91 (7), 78 (6), 77 (9), 65 (9).

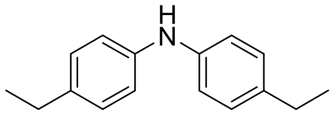

**2f (CAS registry number: 7268-62-4)**

**4-Ethyl-*N*-(4-ethylphenyl)benzenamine (2f**; synthesized from **1f).** MS (70 eV, EI): *m/z* (%): 225 (79) [$M^+$], 226 (15), 211 (16), 210 (100), 195 (12), 194 (6), 180 (6), 98 (9), 77 (6).

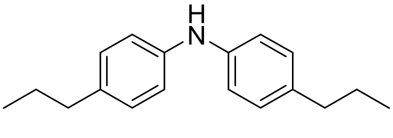

**2g (CAS registry number: 1773485-54-3)**

**4-Propyl-*N*-(4-propylphenyl)benzenamine (2g**; synthesized from **1g).** $^1$H NMR (500 MHz, $CDCl_3$, 25 °C, TMS): $\delta$ = 7.04–7.03 (m, 4H), 6.94–6.92 (m, 4H), 5.46 (brs, 1H), 2.51 (t, $J$ = 7.5 Hz, 4H), 1.59 (sext, $J$ = 7.5 Hz, 4H), 0.93 (t, $J$ = 7.3 Hz, 6H); $^{13}$C{$^1$H} NMR (125 MHz, $CDCl_3$, 25 °C, TMS): $\delta$ = 141.6, 135.4, 129.5, 118.1, 37.6, 25.1, 14.2; MS (70 eV, EI): *m/z* (%): 253 (59) [$M^+$], 254 (12), 225 (18), 224 (100), 195 (15), 194 (6).

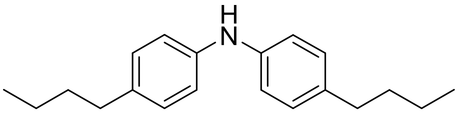

**2h (CAS registry number: 227003-50-1)**

**4-Butyl-*N*-(4-butylphenyl)benzenamine (2h**; synthesized from **1h).** $^1$H NMR (500 MHz, $CDCl_3$, 25 °C, TMS): $\delta$ = 7.05–7.04 (m, 4H), 6.95–6.93 (m, 4H), 5.48 (brs, 1H), 2.53 (t, $J$ = 7.8 Hz, 4H), 1.60–1.54 (m, 4H),

1.39–1.31 (m, 4H), 0.92 (t, $J$ = 7.3 Hz, 6H); $^{13}C\{^1H\}$ NMR (125 MHz, $CDCl_3$, 25 °C, TMS): $\delta$ = 141.6, 135.6, 129.5, 118.1, 35.2, 34.2, 22.7, 14.3; MS (70 eV, EI): *m/z* (%): 281 (47) [$M^+$], 282 (12), 239 (21), 238 (100), 195 (15), 194 (6).

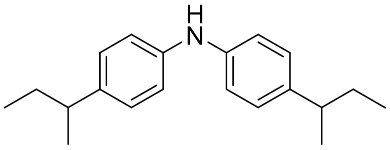

**2i (CAS registry number: 1808252-16-5)**

**4-(1-Methylpropyl)-*N*-[4-(1-methylpropyl)phenyl]benzenamine (2i**; synthesized from **1i).** $^1H$ NMR (500 MHz, $CDCl_3$, 25 °C, TMS): $\delta$ = 7.06–7.04 (m, 4H), 6.97–6.95 (m, 4H), 5.50 (brs, 1H), 2.56–2.49 (m, 2H), 1.58–1.55 (m, 4H), 1.21 (d, $J$ = 7.0 Hz, 6H), 0.83 (t, $J$ = 7.3 Hz, 6H); $^{13}C\{^1H\}$ NMR (125 MHz, $CDCl_3$, 25 °C, TMS): $\delta$ = 141.7, 140.4, 128.0, 118.0, 41.2, 31.6, 22.2, 12.6; MS (70 eV, EI): *m/z* (%): 281 (34) [$M^+$], 282 (8), 266 (7), 253 (22), 252 (100), 223 (6), 222 (12), 112 (7).

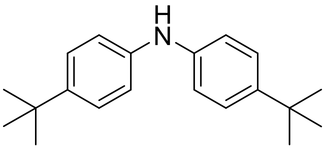

**2j (CAS registry number: 4627-22-9)**

**4-(1,1-Dimethylethyl)-*N*-[4-(1,1-dimethylethyl)phenyl]benzenamine (2j**; synthesized from **1j).** MS (70 eV, EI): *m/z* (%): 281 (40) [$M^+$], 282 (10), 267 (24), 266 (100), 250 (8), 236 (5), 126 (7), 98 (14).

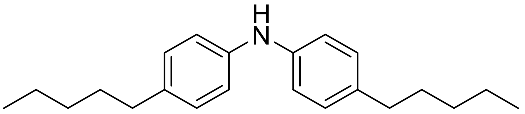

**2k (CAS registry number: 1610428-28-8)**

**4-Pentyl-*N*-(4-pentylphenyl)benzenamine (2k**; synthesized from **1k).** $^1H$ NMR (500 MHz, $CDCl_3$, 25 °C, TMS): $\delta$ = 7.05–7.04 (m, 4H), 6.95–6.93 (m, 4H), 5.49 (brs, 1H), 2.53 (t, $J$ = 7.8 Hz, 4H), 1.58 (quint, $J$ = 7.5 Hz, 4H), 1.35–1.26 (m, 8H), 0.89 (t, $J$ = 6.8 Hz, 6H); $^{13}C\{^1H\}$ NMR (125 MHz, $CDCl_3$, 25 °C, TMS): $\delta$ = 141.6, 135.6, 129.4, 118.1, 35.5, 31.9, 31.7, 22.9, 14.4; MS (70 eV, EI): *m/z* (%): 309 (45) [$M^+$], 310 (11), 253 (21), 252 (100), 208 (5), 195 (15), 194 (6).

Compound data of unsymmetrically substituted diarylamines (**6**)

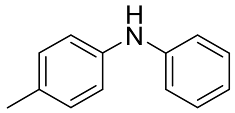

**6a (CAS registry number: 620-84-8)**

**4-Methyl-*N*-phenylbenzenamine (6a**; synthesized from **4b** with **5a**, **4l** with **5b**, **1b** with **5b**, **1c** with **5a, 7b** with **8a**, and **7c** with **8b).** MS (70 eV, EI): *m/z* (%): 183 (100) [$M^+$], 184 (15), 182 (55), 180 (7), 168 (8), 167

(22), 91 (20), 90 (7), 77 (13), 65 (6), 51 (6).

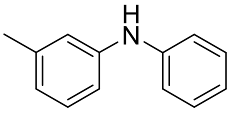

**6b (CAS registry number: 1205-64-7)**

**3-Methyl-*N*-phenylbenzenamine (6b**; synthesized from **4c** with **5a**, **4l** with **5c**, **1b** with **5c**, and **1d** with **5a).** MS (70 eV, EI): *m/z* (%): 183 (100) [$M^+$], 184 (14), 182 (24), 180 (5), 168 (16), 167 (30), 91 (12), 79 (5), 78 (6), 77 (18), 66 (5), 65 (16), 63 (6), 51 (12).

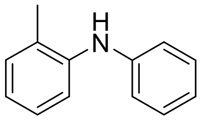

**6c (CAS registry number: 1205-39-6)**

**2-Methyl-*N*-phenylbenzenamine (6c**; synthesized from **4d** with **5a** and **4l** with **5d).** MS (70 eV, EI): *m/z* (%): 183 (100) [$M^+$], 184 (14), 182 (59), 181 (5), 180 (20), 168 (18), 167 (30), 166 (6), 165 (10), 117 (5), 106 (23), 104 (6), 91 (17), 90 (5), 84 (5), 78 (6), 77 (14), 65 (9), 51 (9).

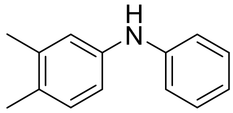

**6d (CAS registry number: 17802-36-7)**

**3,4-Dimethyl-*N*-phenylbenzenamine (6d**; synthesized from **4e** with **5a**, **4l** with **5e**, and **1b** with **5e).** $^1$H NMR (500 MHz, $CDCl_3$, 25 °C, TMS): $\delta$ = 7.22–7.18 (m, 2H), 7.01–6.96 (m, 3H), 6.86–6.81 (m, 3H), 5.49 (brs, 1H), 2.19 (s, 3H), 2.19 (s, 3H); $^{13}$C{$^1$H} NMR (125 MHz, $CDCl_3$, 25 °C, TMS): $\delta$ = 144.3, 140.9, 137.8, 130.6, 129.9, 129.6, 120.6, 120.4, 117.1, 116.5, 20.2, 19.3; MS (70 eV, EI): *m/z* (%): 197 (100) [$M^+$], 198 (16), 196 (39), 182 (25), 181 (12), 180 (15), 167 (6), 105 (8), 98 (5), 91 (5), 90 (7), 77 (11).

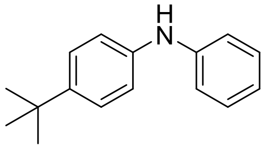

**6e (CAS registry number: 4496-49-5)**

**4-(1,1-dimethylethyl)-*N*-phenylbenzenamine (6e**; synthesized from **4f** with **5a**, **4l** with **5i**, **1b** with **5i**, and **1j** with **5a).** MS (70 eV, EI): *m/z* (%): 225 (48) [$M^+$], 226 (9), 211 (17), 210 (100), 195 (7), 194 (6), 182 (5), 180 (7), 168 (5), 167 (5), 92 (9), 91 (8), 90 (9).

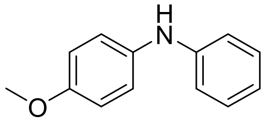


**6f (CAS registry number: 1208-86-2)**

**4-Methoxy-*N*-phenylbenzenamine (6f**; synthesized from **4g** with **5a** and **7a** with **8a).** MS (70 eV, EI): *m/z*

(%): 199 (86) [$M^{+}$], 200 (13), 185 (15), 184 (100), 167 (5), 155 (5), 154 (9), 129 (16), 128 (13), 77 (12), 51 (7).

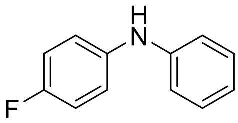

**6g (CAS registry number: 330-83-6)**

**4-Fluoro-*N*-phenylbenzenamine (7g**; synthesized from **4h** with **5a).** MS (70 eV, EI): *m/z* (%): 187 (83) [$M^{+}$], 188 (11), 186 (32), 185 (16), 170 (13), 169 (100), 168 (53), 167 (27), 166 (6), 93 (6), 84 (13), 77 (11), 66 (12), 65 (8), 51 (12).

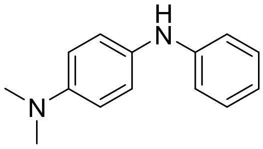

**6h (CAS registry number: 3586-00-3)**

***N*[1],*N*[1]-Dimethyl-*N*[4]-phenyl-1,4-benzenediamine (6h**; synthesized from **4i** with **5a).** MS (70 eV, EI): *m/z* (%): 212 (100) [$M^{+}$], 213 (16), 211 (11), 198 (8), 197 (38), 196 (9), 170 (6), 169 (7), 168 (6), 167 (14), 106 (7), 105 (5), 92 (17), 77 (6).

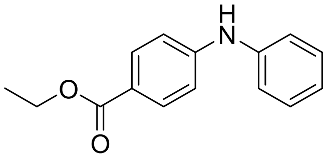

**6i (CAS registry number: 64678-66-6)**

**Ethyl 4-(phenylamino)benzoate (6i**; synthesized from **4j** with **5a** and **4l** with **5j).** MS (70 eV, EI): *m/z* (%): 241 (100) [$M^{+}$], 242 (18), 214 (6), 213 (37), 197 (12), 196 (72), 169 (7), 168 (17), 167 (39), 166 (7), 98 (5), 84 (9), 77 (5).

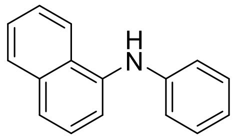

**6j (CAS registry number: 90-30-2)**

***N*-Phenyl-1-naphthalenamine (6j**; synthesized from **4k** with **5a).** MS (70 eV, EI): *m/z* (%): 219 (100) [$M^{+}$], 220 (17), 218 (50), 217 (38), 216 (8), 115 (10), 110 (5), 109 (17).

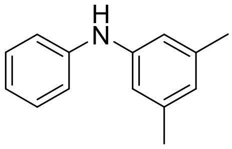

**6k (CAS registry number: 51786-49-3)**

**3,5-Dimethyl-*N*-phenylbenzenamine (6k**; synthesized from **4l** with **5f** and **1b** with **5f).** MS (70 eV, EI): *m/z* (%): 197 (100) [$M^{+}$], 198 (16), 196 (20), 182 (12), 181 (24), 180 (14), 167 (10), 98 (5), 90 (6), 77 (10).

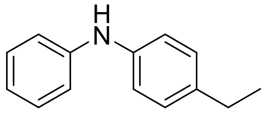

**6l (CAS registry number: 32804-22-1)**

**4-Ethyl-*N*-phenylbenzenamine (6l**; synthesized from **4l** with **5g** and **1b** with **5g).** MS (70 eV, EI): *m/z* (%): 197 (58) [$M^+$], 198 (10), 183 (15), 182 (100), 180 (6), 168 (5), 167 (11), 90 (6), 77 (9).

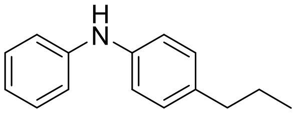

**6m (CAS registry number: 38158-63-3)**

***N*-Phenyl-4-propylbenzenamine (6m**; synthesized from **4l** with **5h** and **1b** with **5h).** $^1$H NMR (500 MHz, $CDCl_3$, 25 °C, TMS): $\delta$ = 7.24–7.20 (m, 2H), 7.07–7.06 (m, 2H), 7.00–6.98 (m, 4H), 6.86 (t, $J$ = 7.3 Hz, 1H), 5.56 (brs, 1H), 2.52 (t, $J$ = 7.5 Hz, 2H), 1.61 (sext, $J$ = 7.4 Hz, 2H), 6.94 (t, $J$ = 7.3 Hz, 3H); $^{13}$C{$^1$H} NMR (125 MHz, $CDCl_3$, 25 °C, TMS): $\delta$ = 144.1, 140.8, 136.1, 130.0, 119.4, 118.5, 117.7, 116.8, 37.7, 25.0, 14.2; MS (70 eV, EI): *m/z* (%): 211 (41) [$M^+$], 212 (7), 183 (15), 182 (100), 180 (6), 167 (8), 77 (7).

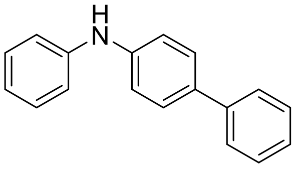

**6n (CAS registry number: 32228-99-2)**

***N*-Phenyl-[1,1'-biphenyl]-4-amine (6n**; synthesized from **4l** with **5k** and **1b** with **5k).** $^1$H NMR (500 MHz, $CDCl_3$, 25 °C, TMS): $\delta$ = 7.52–7.00 (m, 14H), 5.59 (brs, 1H); $^{13}$C{$^1$H} NMR (125 MHz, $CDCl_3$, 25 °C, TMS): $\delta$ = 143.1, 142.8, 141.1, 133.9, 129.7, 129.0, 128.2, 126.9, 126.8, 121.4, 118.3, 118.0; MS (70 eV, EI): *m/z* (%): 245 (100) [$M^+$], 246 (21), 244 (17), 243 (5), 167 (5).

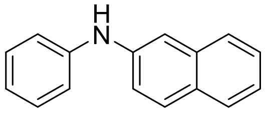

**6o (CAS registry number: 135-88-6)**

***N*-Phenyl-2-naphthalenamine (6o**; synthesized from **4l** with **5l).** MS (70 eV, EI): *m/z* (%): 219 (100) [$M^+$], 220 (18), 218 (39), 217 (23), 216 (5), 115 (8), 109 (9).

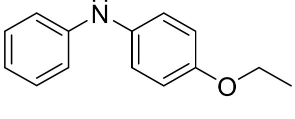

**6p (CAS registry number: 1020-54-8)**

**4-Ethoxy-*N*-phenylbenzenamine (6p**; synthesized from **1b** with **5m).** MS (70 eV, EI): *m/z* (%): 213 (78) [$M^+$], 214 (13), 185 (27), 184 (100), 154 (6), 129 (11), 128 (9), 77 (7).

**Table S1** Acceptorless dehydrogenative aromatization of **1a** using various catalysts[a]

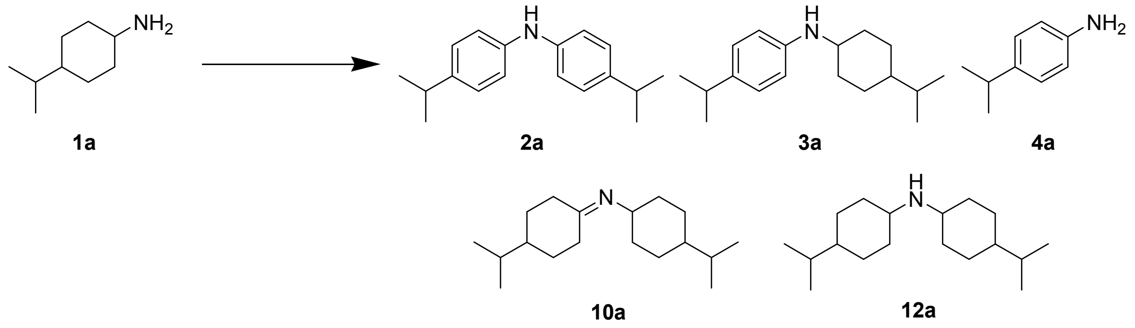

| Entry | Catalyst | Conv. [%] | Yield [%] | | | | |
|---|---|---|---|---|---|---|---|
| | | **1a** | **2a** | **3a** | **4a** | **10a** | **12a** |
| 1 | Au–Pd/$TiO_2$ | >99 | 54 | 36 | 4 | <1 | <1 |
| 2[b,c] | Au–Pd/$TiO_2$ | >99 | 88 | 3 | 3 | <1 | <1 |
| 3[d] | Au/$TiO_2$ | 34 | <1 | <1 | <1 | 1 | 1 |
| 4[b,d] | Au/$TiO_2$ | 44 | <1 | <1 | <1 | 5 | 6 |
| 5[e] | Pd/$TiO_2$ | 95 | 30 | 36 | 23 | <1 | <1 |
| 6[b,e] | Pd/$TiO_2$ | >99 | 44 | 25 | 24 | <1 | <1 |
| 7[f] | Au/$TiO_2$ + Pd/$TiO_2$ | >99 | 44 | 43 | 4 | <1 | <1 |
| 8[b,f] | Au/$TiO_2$ + Pd/$TiO_2$ | >99 | 69 | 15 | 4 | <1 | <1 |
| 9[g] | Au–Pd/$TiO_2$ | >99 | 43 | 52 | 3 | <1 | <1 |
| 10[h] | Au–Pd/$TiO_2$ | >99 | 46 | 25 | 21 | <1 | <1 |
| 11[i] | Au–Pd/$TiO_2$ | >99 | 40 | 21 | 38 | <1 | <1 |
| 12 | Au–Pd/$Al_2O_3$ | >99 | 44 | 47 | 2 | <1 | <1 |
| 13 | Au–Pd/$CeO_2$ | >99 | 44 | 45 | 10 | <1 | <1 |
| 14 | Au–Pd/MgO | >99 | 29 | 61 | 8 | <1 | <1 |
| 15 | Au–Pd/$SiO_2$ | >99 | 3 | 74 | 1 | <1 | 10 |

[a]Reaction conditions: catalyst (total metal (Au + Pd): 2.5 mol%), **1a** (1.0 mmol), mesitylene (2 mL), 160 °C, Ar (1 atm), 6 h. Yields were determined by GC analysis using *n*-hexadecane as an internal standard. [b]24 h. [c]Co-production of hydrogen gas (2.8 mmol) and ammonia gas (0.3 mmol). [d]Au: 1.45 mol%. [e]Pd: 1.05 mol%. [f]A physical mixture of Au/$TiO_2$ and Pd/$TiO_2$ (Au: 1.45 mol%, Pd: 1.05 mol% with respect to **1a**). [g]Au/Pd = 76/24 (mol/mol). [h]Au/Pd = 35/65 (mol/mol). [i]Au/Pd = 14/86 (mol/mol).

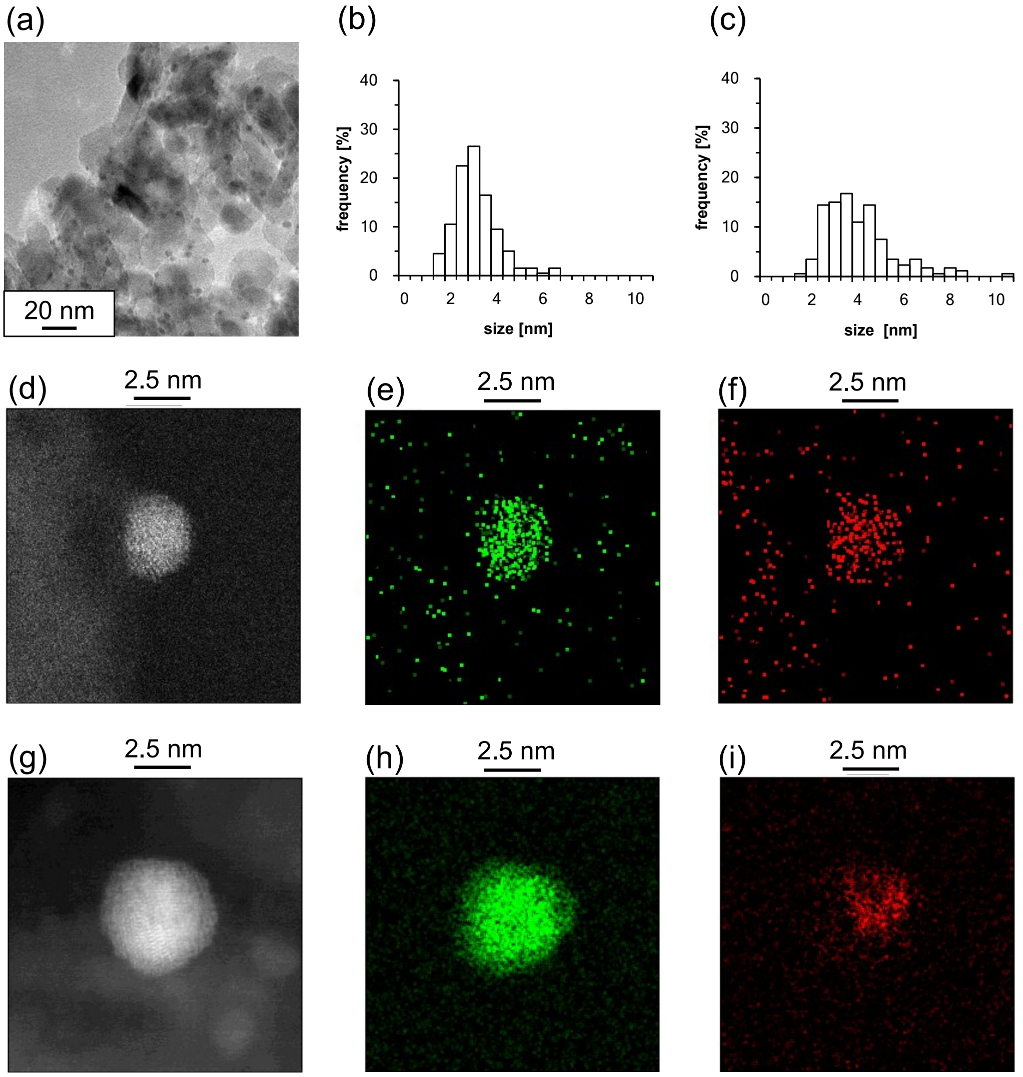


**Fig. S1** (a) TEM image of Au–Pd/$TiO_2$. (b) The size distribution of bimetallic nanoparticles (average: 3.3 nm, σ: 1.0 nm). (c) The size distribution of bimetallic nanoparticles after the third reuse experiment (average: 4.4 nm, σ: 1.9 nm). The size distributions were determined using 200 particles. (d) HAADF-STEM image of Au–Pd/$TiO_2$. (e) EDS image (Au element) of Au–Pd/$TiO_2$. (f) EDS image (Pd element) of Au–Pd/$TiO_2$. (g) HAADF-STEM image of Au–Pd/$TiO_2$ used after the third reuse experiment. (h) EDS image (Au element) of Au–Pd/$TiO_2$ used after the third reuse experiment. (i) EDS image (Pd element) of Au–Pd/$TiO_2$ used after the third reuse experiment.

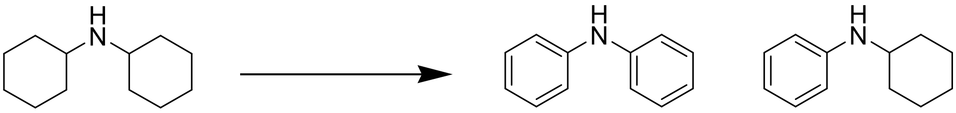

**12b** **2b** **3b**

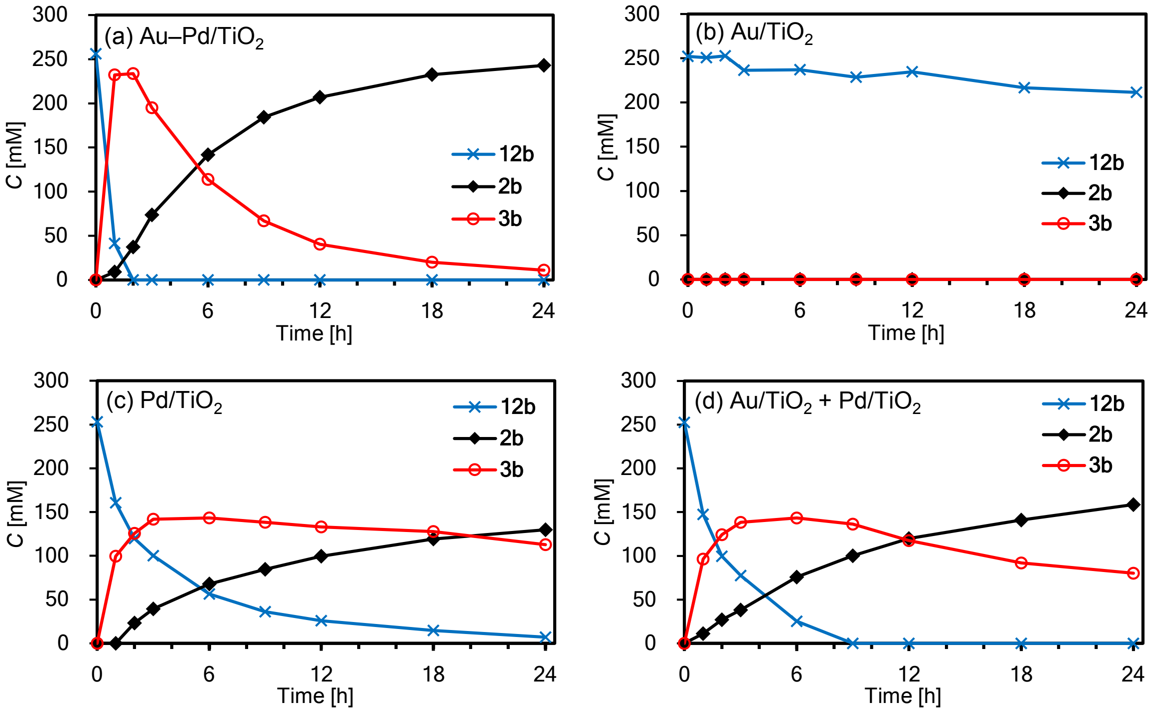


**Fig. S2** Reaction profiles for the acceptorless dehydrogenative aromatization of **12b**. Reaction conditions: Catalyst (Au: 2.9 mol%, Pd: 2.1 mol%), **12b** (0.5 mmol), mesitylene (2 mL), 160 °C, Ar (1 atm). Yields were determined by GC analysis using *n*-hexadecane as an internal standard. (a) Au–Pd/$TiO_2$. (b) Au/$TiO_2$. (c) Pd/$TiO_2$. (d) Au/$TiO_2$ + Pd/$TiO_2$ (physical mixture).

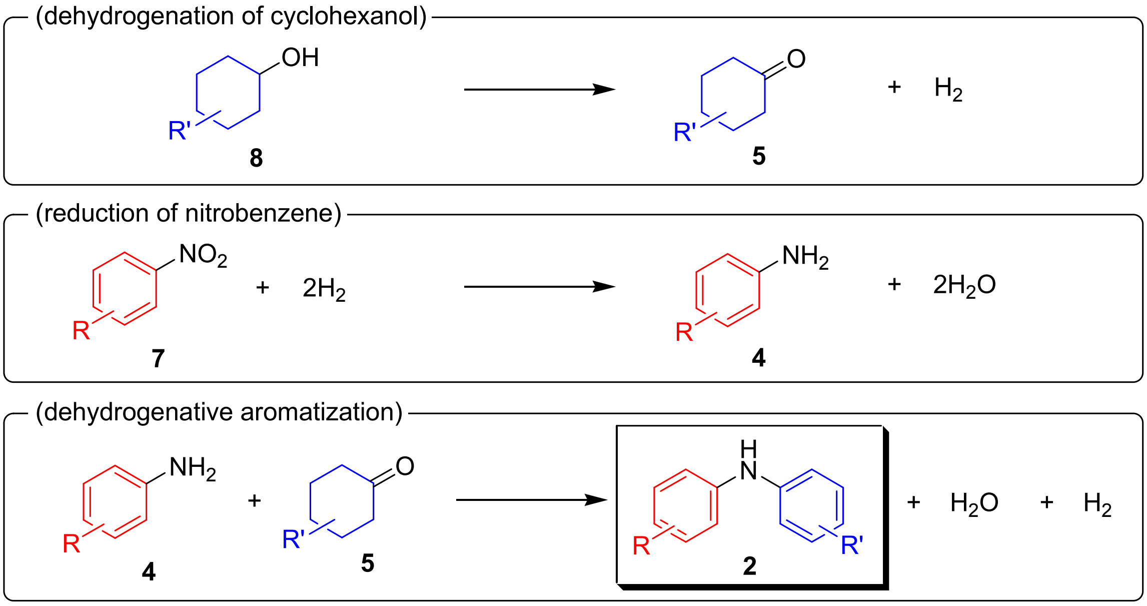


**Fig. S3** Proposed reaction pathways for the Au–Pd/$TiO_2$-catalyzed acceptorless dehydrogenative aromatization of nitrobenzenes and cyclohexanones. Initially, dehydrogenation of cyclohexanol proceeds to give the cyclohexanone. Then, nitrobenzene is reduced to the aniline by hydrogen. Finally, dehydrogenative aromatization of cyclohexanone and anilines proceeds to give the corresponding diarylamine. The reduction of nitrobenzene utilize the hydrogen (or transiently formed metal hydride spices directly) formed in the dehydrogenation of cyclohexanol and dehydrogenative aromatization steps.

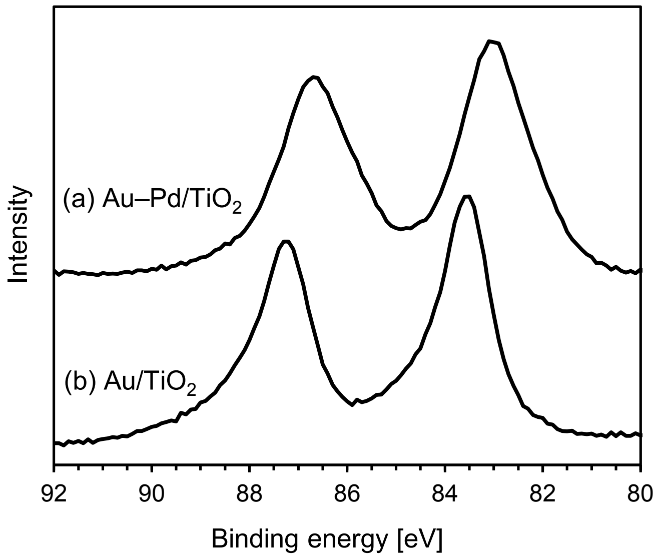


**Fig. S4** XPS spectra of (a) Au–Pd/$TiO_2$ and (b) Au/$TiO_2$ around Au 4f components. The two peaks in each spectrum observed around 83 eV and 87 eV are attributed to Au $4f_{7/2}$ and $4f_{5/2}$, respectively. The Au 4f peaks of Au–Pd/$TiO_2$ were observed at more negative binding energies in comparison with those of Au/$TiO_2$. These significant negative shifts indicate the net electron-transfer from palladium to gold by alloying. Such phenomena have frequently observed for Au–Pd alloy nanoparticles catalysts (see ref. 10). We also attempted to confirm the peak shifts of Pd 3d components in Au–Pd/$TiO_2$. However, it was very difficult to confirm that because the peak due to Pd $3d_{5/2}$ was overlapped with that of Au $4d_{5/2}$ and the intensity of the peak attributed to Pd $3d_{3/2}$ was very low.
